# Control-Relevance-Aware Sensor Integrity in a Position-Controlled DC Motor: Surrogate Evaluation and an Exploratory Direct-Simulation Crossover Test

**DOI:** 10.3390/s26216818

**Published:** 2026-10-28

**Authors:** Atef M. Ghaleb, Ali S. Allahloh, Mohammad Sarfraz, Abdalla Alrashdan, Sobhi Mejjaouli, Mohammed A. H. Ali, Fahad M. Alqahtani, Adel Al-Shayea

**Affiliations:** 1 Department of Industrial Engineering, College of Engineering and Advanced Computing, Alfaisal University, Riyadh 11533, Saudi Arabia; aghaleb@alfaisal.edu (A.M.G.); aalrshdan@alfaisal.edu (A.A.); smejjaouli@alfaisal.edu (S.M.); 2 Department of Electrical Engineering, Zakir Husain College of Engineering and Technology (ZHCET), Aligarh Muslim University, Aligarh 202002, India; msarfraz@zhcet.ac.in; 3 Department of Mechanical Engineering, Faculty of Engineering, Universiti Malaya, Kuala Lumpur 50603, Malaysia; hashem@um.edu.my; 4 Department of Industrial Engineering, College of Engineering, King Saud University, P.O. Box 800, Riyadh 11421, Saudi Arabia; afahad@ksu.edu.sa

**Keywords:** fault-tolerant control, sensor integrity, control relevance, robust filtering, sensor fusion, uncertainty calibration, domain shift, embedded control, motor drive, reproducible simulation

## Abstract

**Highlights:**

**What are the main findings?**

- CRASI improves selective tracking relative to the original residual-plus-uncertainty comparator, but the benefit is fault- and comparator-dependent rather than universal.
- Revision diagnostics expose comparator dependence, simultaneous-fault blindness, current self-masking, and an inactive position-uncertainty derating path.

**What are the implications of the main findings?**

- Tracking gains against an aggressive residual comparator must be distinguished from active fault accommodation and from safety improvement.
- Fresh pre-specified direct Proteus/HIL/physical validation is required before transfer or deployment claims.

**Abstract:**

Control-relevance-aware sensor integrity (CRASI) combines heuristic relevance, common-evidence subtraction, finite trust retention and bias tracking. We evaluate an internally specified 2520-episode DC-motor surrogate campaign calibrated from Proteus characterization. In the held-out modeled 70% load domain, CRASI reduced integral absolute error (IAE) versus residual-plus-uncertainty by 37.6% for encoder bias, 19.9% for drift, 15.9% for current under-read and 4.4% for current bias. Fixed control performed better for drift/current bias, under-read matched fixed, and dropouts were effectively null; adaptive Huber was 2.0% better for drift. Revision diagnostics identify comparator-dependent attribution, simultaneous-fault blindness, current self-masking, inactive position-uncertainty derating, and no hard-safety superiority. Matched-observer, detection/recovery, parameter-sensitivity and guard-stress analyses therefore restrict mechanism claims. A revision-era direct Proteus audit is not treated as validation because its acquisition/analysis gates were amended after partial outcome inspection and protection trips materially affected several 50% cells; the audit is retained only as a transparent research record and is not used as evidence for the manuscript conclusions. Findings support selective tracking comparisons under modeled sensor/plant confounding, while fresh pre-specified direct Proteus/HIL/physical validation remains necessary.

## 1. Introduction

Sensor faults are a central reliability problem in closed-loop electric drives because measurements influence state estimation and actuator commands. Classical model-based fault diagnosis provides residual-generation and isolation tools [1,2,3], while recent reviews and drive studies cover observer-based diagnosis, analytical redundancy, signal reconstruction, and fault-tolerant reconfiguration [4,5,6,7,8,9]. Parameter-robust EKF compensation [10], observer-bank reconfiguration [11], unified PMSM FTC [12], and rotor-flux-observer diagnosis with laboratory validation [13] address architectures in which current feedback directly affects torque/flux control. The present testbed instead has a PD position loop: position and estimated speed come from one quadrature encoder, and current enters only a multiplicative constraint envelope. It has no independently sensed speed channel, cascaded current controller, or FOC loop. Current-sensor findings therefore concern this particular envelope and cannot establish performance in those conventional drive architectures.

A different difficulty arises before replacement is justified. In a closed loop, residuals respond not only to sensor faults but also to load changes, actuator variation, model error, operating-point transitions, and controller action. Recent reconfigurable-drive studies explicitly note that coupled plant faults can contaminate model-based sensor residuals and trigger incorrect diagnoses [11]. Parameter-robust observers reduce this sensitivity [10], but the usual decision remains diagnosis-centric: sufficiently abnormal residual evidence causes down-weighting, replacement, or switching. That logic can be harmful when the measurement is imperfect yet still more useful to the controller than the nominal model under plant shift.

The coupling between diagnosis and control has long motivated integrated FTC. Du et al. formalized a trade-off between fault detectability and closed-loop performance [14], while control-aware fault detection has incorporated control actions into learned dynamic surrogate models for non-stationary processes [15]. In parallel, uncertainty-aware diagnosis increasingly emphasizes calibrated confidence rather than point decisions [16]; conformal methods provide distribution-free uncertainty quantification under stated assumptions, while recent industrial studies address fault diagnosis and time-series distribution shift directly [17,18,19]. These strands leave a specific embedded-control gap: neither a large residual nor a wide uncertainty interval directly answers whether distrust of a particular sensor will improve the present closed-loop decision. Recent work reinforces the need to separate calibration reliability from transferability under changing operating conditions. Cross-asset residual conformal monitoring has emphasized asset-disjoint evaluation and explicit false-alarm auditing under distribution shift [20], while physics-guided bearing diagnosis under unit and rotational-speed variability shows that calibrated false-alarm behavior can coexist with degraded diagnostic sensitivity and localization [21].

This paper examines control-relevance-aware sensor integrity (CRASI) in a position-controlled DC-motor surrogate. CRASI combines empirical prediction scales, partial common-evidence subtraction, heuristic channel relevance, finite-measurement trust floors, and bias tracking. The original comparison asks whether this combined design improves tracking and nuisance rejection relative to a normalized residual-plus-uncertainty baseline under modeled load shift. It does not by itself isolate relevance from trust-floor and bias-tracker effects; matched revision comparators and existing component ablations address that distinction. The objective is tracking behavior under sensor/plant confounding, with rejection and finite-measurement action reported separately.

The contributions are an explicit lightweight integrity rule, the original paired 2520-episode surrogate comparison, and an audit that restricts mechanism and safety claims. Existing Huber and alternate-load studies and revision diagnostics are exploratory. A revision-era direct Proteus audit is retained only as a transparency record: because its original acquisition criteria were not met by the complete allocation and analysis gates were amended after partial outcome inspection, it is not used as validation or as support for the manuscript conclusions.

**Figure 1:**
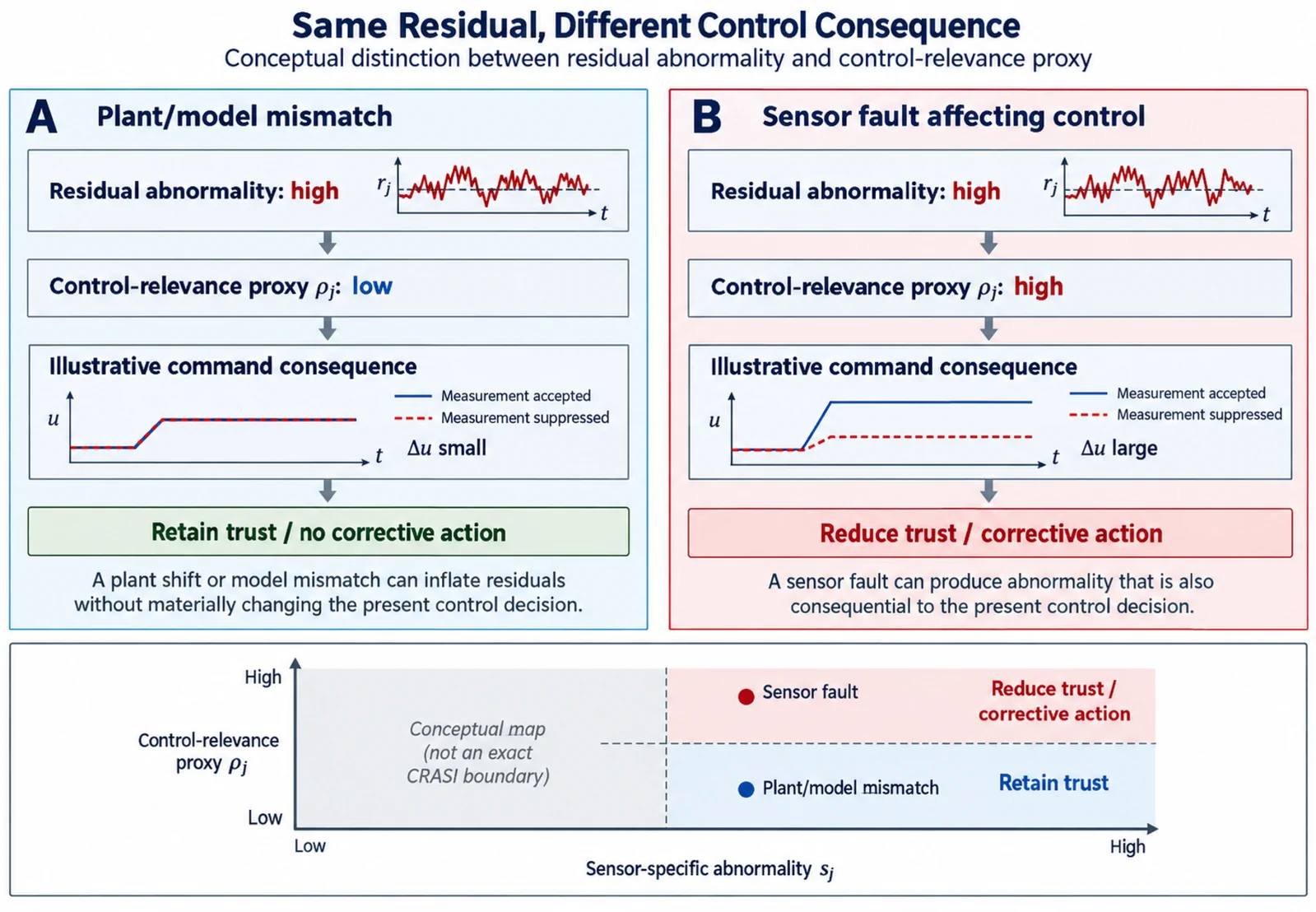
Conceptual motivation for control-relevance-aware sensor integrity. Similar residual abnormality can arise from plant/model mismatch or from a sensor fault, yet the consequences for the control decision may differ substantially. (**Panel A**) depicts plant/model mismatch with high residual abnormality but low control relevance, leading to retained trust/no corrective action; (**Panel B**) depicts a sensor fault with high residual abnormality and high control relevance, leading to reduced trust/corrective action. Gray downward arrows indicate the conceptual progression from residual evidence to relevance, command consequence, and integrity action. Blue denotes the measurement-accepted/retain-trust path and the plant/model-mismatch case; red denotes the measurement-suppressed/reduce-trust path and the sensor-fault case. In the conceptual map, the blue shaded region corresponds to retain trust and the red shaded region corresponds to reduce trust/corrective action. CRASI combines sensor-specific abnormality $s_{j}$ with a heuristic control-relevance proxy $\rho_{j}$ to moderate finite-sensor trust and corrective action. The command trajectories and decision regions are schematic illustrations, not study data or an exact numerical representation of the CRASI action boundary.

Figure 1 illustrates the conceptual distinction between residual abnormality and decision relevance. The control-relevance quantity is a heuristic operating-point proxy rather than the actual command counterfactual; a tracking benefit against a more aggressive comparator therefore does not by itself prove active fault compensation.

## 2. Materials and Methods

### 2.1. Study Boundary, Internal Specification, and Evidence Provenance

This study has two evidentiary layers: preserved Proteus VSM characterization used for calibration, and the original final host-side surrogate fault campaign. Revision diagnostics interrogate the same archived implementation and add clearly labeled exploratory host-side sensitivity studies. A separate revision-era direct Proteus audit was also attempted; because the complete allocation did not satisfy the original strict acquisition criteria and the analysis gates were amended after partial outcome inspection, that audit is excluded from the manuscript evidentiary basis and retained by the authors only for transparency.

The supplied protocol is identified as CRASI_PHASE5_FINAL_V1 and has SHA-256 digest e0d779d73f427aed70d4c8a6e8372eaafdfb8dc58e08db887ce8ccfc23789983. It specifies domains, seeds, fault windows, controller parameters, thresholds, endpoints, and statistical procedures. Its internal metadata states a freeze time of 11 September 2026 at 18:45 UTC; the current bytes match the archived digest. However, no independently verifiable timestamp, external deposit, or repository history in the supplied package proves that this record preceded inspection of the final results. We therefore describe the original analysis as internally specified/hash-recorded and reproducible, not externally verified prospective registration. Statistical significance below is conditional on the supplied analysis specification; the chronology of its specification is not independently established.

This distinction is central to interpretation: the final Phase-5 episodes are *not* claimed to have run in Proteus or on physical hardware. Historical Proteus results are used as calibration evidence; the final causal comparisons are surrogate-domain experiments.

### 2.2. Embedded Co-Simulation Characterization Used to Build the Surrogate

The historical testbed used a Proteus 9.1 SP2 MOTOR-ENCODER plant, an ATmega2560 controller, quadrature encoder feedback, an ACS712-05B current sensor model, and an L298 dual full-bridge driver. The ATmega2560 is an 8-bit AVR microcontroller with 256 kB flash, 8 kB SRAM and a 10-bit ADC [22]; the ACS712 provides an analog current-proportional output [23]; and the L298 is a dual full-bridge motor driver [24]. The frozen historical configuration used 960 decoded encoder counts/revolution, a 100 Hz control and telemetry rate, and a command range of [−192,192] PWM units. No invalid quadrature transitions or scheduler deadline misses were recorded in the selected characterization evidence.

Current conversion was based on an 11-point calibration,

(1) $$V_{\mathrm{ADC}}=V_{0}+S_{I}I,$$

with $V_{0}=2.50270$ V, $S_{I}=0.198927$ V/A, $R^{2}=0.999855$, and calibration RMSE 1.639 mV. The experimentally verified breakaway/sustain dead-zone magnitude was 12 PWM units. At 50% virtual load, the preserved operating-point table spans 32–192 PWM; the corresponding absolute mean speeds range from 9.09 to 1253.54 encoder counts/s and mean current magnitude from 1.30 mA to 264.06 mA. These preserved operating points are used directly rather than being re-fitted into a high-order motor model.

### 2.3. Empirical Plant and Sensor Surrogate

Let $u_{k}$ denote the signed PWM command and *L* the dimensionless load fraction. The steady-speed and steady-current maps are obtained by linear interpolation of the 50% load characterization table:

(2) $$\begin{array}{cc} \bar{v}\left(u,L\right) & =\mathrm{sgn}\left(u\right)\;I_{v}\left(|u|\right)\;s_{v}\left(L\right), \end{array}$$

(3) $$\begin{array}{cc} \bar{i}\left(u,L\right) & =\mathrm{sgn}\left(u\right)\;I_{i}\left(|u|\right)\;s_{i}\left(L\right), \end{array}$$

where $I_{v}$ and $I_{i}$ are empirical lookup interpolants. The internally specified load continuations are

(4) $$s_{v}\left(L\right)=\mathrm{clip}\;\left(\frac{1-L}{1-0.50},0.15,1.8\right),\;s_{i}\left(L\right)=\mathrm{clip}\;\left(1+1.15(L-0.50),0.7,1.8\right).$$

These continuations are engineering stress-model assumptions, not measured 70% load transients. The current update below is memoryless conditional on PWM and load; no electrical transient, inductance, back-EMF coupling, or thermal driver model is identified from the supplied steady-state points. These omissions are material when evaluating current-sensor safety.

The 10 ms state update uses a first-order speed response:

(5) $$\begin{array}{cc} v_{k+1} & =av_{k}+\left(1-a\right)\bar{v}\left(u_{k},L\right)+w_{k}^{v}, \end{array}$$

(6) $$\begin{array}{cc} q_{k+1} & =q_{k}+\frac{\Delta t}{2}\left(v_{k}+v_{k+1}\right), \end{array}$$

(7) $$\begin{array}{cc} i_{k+1} & =\bar{i}\left(u_{k},L\right)+w_{k}^{i}, \end{array}$$

where a=exp(−Δt/0.343) when driven and a=exp(−Δt/1.21) when coasting. Encoder measurements are quantized to whole decoded counts after domain-specific threshold jitter. Current is quantized using the 10-bit ADC and calibrated ACS712 slope, giving an effective current quantization step of approximately 24.57 mA. Process and measurement noise parameters were fixed independently for each domain in the protocol.

### 2.4. Retrospective Surrogate Cross-Check and Post-Primary Load Sensitivity

Two analyses added after inspection of the original primary results challenge the principal surrogate assumption without altering the original primary results. First, the protocol surrogate—constructed from the 50% load lookup table—was retrospectively compared with the preserved Proteus operating points at 10, 20 and 30% load. Speed mean absolute errors were 37.9, 27.3 and 15.0 counts/s (sMAPE 13.4, 10.9 and 8.5%), respectively. Current mean absolute errors were 3.10, 5.92 and 5.26 mA; percentage current error is less informative because several measured currents are near zero. This cross-check is not independent validation because the current-load continuation was informed by the 10–50% characterization campaign, but it quantifies consistency with preserved operating points not used as the 50% lookup table.

Second, a deliberately different 70% load plant was constructed after the primary analysis. At each PWM level, speed and current were linearly regressed against the actual 10, 20, 30 and 50% Proteus measurements and extrapolated to 70%; interpolation was then performed across PWM. The controller/observer retained the original 50% nominal map and all controller, integrity, uncertainty, seed, fault and reference parameters were unchanged. This *post-primary exploratory sensitivity analysis* therefore asks whether the observed comparison depends on the single analytic load-scaling law used in the primary surrogate. It is reported as robustness evidence, not as a second original-analysis experiment and not as measured 70% load Proteus data.

### 2.5. Controller and Safety Envelope

Define the unsaturated PD demand as

(8) $${\tilde{u}}_{k}=K_{p}\left(r_{k}-{\hat{q}}_{k}\right)-K_{d}{\hat{v}}_{k}.$$

With $K_{p}=3.0$, $K_{d}=0.12$, dead-zone compensation $d_{z}=12$ PWM units, and ϵ=0.5, the compensated bounded command is

(9) $$u_{k}^{\mathrm{PD}}={\mathrm{sat}}_{192}\;\left[{\tilde{u}}_{k}+d_{z}\;\mathrm{sgn}\left({\tilde{u}}_{k}\right)\;1_{|{\tilde{u}}_{k}|>\epsilon }\right].$$

All methods use the same current safety envelope. Let $m_{i,k}=\left|{\hat{i}}_{k}\right|+U_{i,k}$. Its multiplicative current factor is

(10) $$\phi_{i}\left(m\right)=\left\{\begin{array}{cc} 1, & m\leq I_{\mathrm{warn}},\\ \frac{I_{\mathrm{soft}}-m}{I_{\mathrm{soft}}-I_{\mathrm{warn}}}, & I_{\mathrm{warn}}<m<I_{\mathrm{soft}},\\ 0, & m\geq I_{\mathrm{soft}}, \end{array}\right.$$

where $I_{\mathrm{warn}}=0.18$ A and $I_{\mathrm{soft}}=0.30$ A. The hard evaluation guard is 0.90 A. Position excursions beyond ±260 counts are counted as guard violations, while the surrogate has a separate ±300-count truth boundary. For uncertainty-aware methods, the position-uncertainty derating factor $\phi_{q}\left(U_{q}\right)$ defined below additionally multiplies $u_{k}^{\mathrm{PD}}$; thus, $u_{k}=\phi_{i}\phi_{q}u_{k}^{\mathrm{PD}}$ before the final command-limit clip. Fixed and residual-only methods use $\phi_{q}=1$.

### 2.6. Calibration Partition and Uncertainty Baselines

Three healthy calibration domains (loads 42, 50 and 58%) and five seeds per domain were evaluated for 30 s each, producing 45,000 one-step samples per channel. Calibration data never use final seeds. For absolute one-step state errors $e_{j}$, the standard split-conformal upper-order statistic was used as an empirical calibration rule,

(11) $$Q_{1-\alpha }=e_{\left(\lceil (n+1)(1-\alpha )\rceil \right)},\;\alpha =0.05.$$

The baseline uncertainty state was stored as $U_{j}=Q_{0.95}/1.96$, so the reported empirical coverage criterion is $|e_{j}|\leq 1.96U_{j}$. The calibrated baselines were $U_{q}=0.5$ count and $U_{i}=0.012285$ A; corresponding residual baselines were 1.0 count and 0.024570 A. The factor 1.96 is only a scale convention; no Gaussian error model is assumed. Because samples within each episode are serially dependent, and because the online uncertainty states adapt after calibration while the held-out domain is distribution-shifted, the exchangeability conditions required for a formal split-conformal finite-sample guarantee are not asserted here. The order statistic is therefore used for transparent empirical calibration rather than as a fault-conditional or domain-shift coverage theorem; recent industrial conformal studies likewise emphasize the consequences of temporal dependence and distribution shift for coverage and fault-diagnosis confidence [18,19].

### 2.7. Control-Relevance-Aware Sensor Integrity

**Figure 2:**
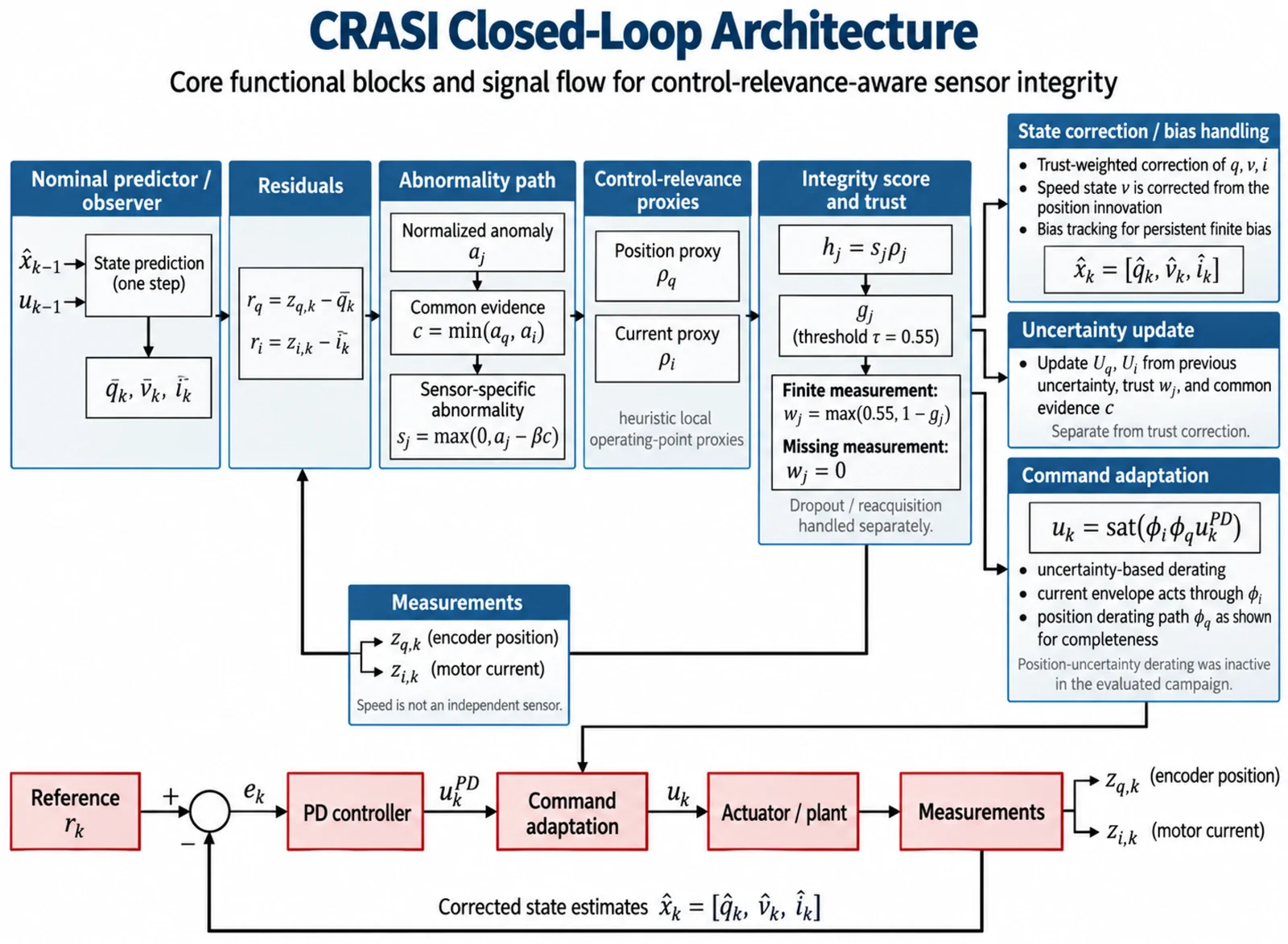
Closed-loop CRASI architecture. Encoder position and motor current are the two measurement channels; the speed state is corrected from the encoder-position innovation rather than from an independent speed sensor. Arrows indicate signal flow through the observer, residual/evidence processing, integrity/trust computation, state correction, command adaptation, actuator/plant, and feedback paths. Blue blocks denote observer, integrity, uncertainty, and state-correction processing, whereas red blocks denote the reference/controller/actuator closed-loop path. Residual abnormality is converted to sensor-specific evidence and combined with heuristic control-relevance proxies to obtain the integrity score and finite-measurement trust; missing measurements use a separate dropout/reacquisition path. Trust-weighted state correction and bias tracking are distinct from uncertainty updates, while uncertainty-based derating acts through the command envelope. The diagram summarizes implementation flow; equations and thresholds are defined in the text, and the original position-uncertainty derating pathway remained inactive in the evaluated campaign.

Figure 2 summarizes the method. A nominal one-step predictor produces $\left(q_{k}^{-},v_{k}^{-},i_{k}^{-}\right)$ from the previous estimate and command. Residuals are

(12) $$r_{q}=z_{k}^{q}-q_{k}^{-},\;r_{i}=z_{k}^{i}-i_{k}^{-}.$$

Each residual is mapped to normalized anomaly evidence $a_{j}\in \left[0,1\right]$ using a piecewise-linear interval from its calibrated residual baseline $b_{j}$ to $5b_{j}$:

(13) $$a_{j}={\mathrm{clip}}_{\left[0,1\right]}\left(\frac{|r_{j}|-b_{j}}{4b_{j}}\right).$$

Missing measurements are assigned $a_{j}=1$ and handled by dedicated dropout and reacquisition logic.

To avoid assigning plant mismatch to one sensor merely because both channels disagree with the nominal model, common evidence is

(14) $$c=\min\left(a_{q},a_{i}\right),\;s_{j}=\max\left(0,a_{j}-\beta c\right),\;\beta =0.5.$$

The terms $s_{j}$ are sensor-specific evidence after partial common-mode subtraction.

The relevance quantities are heuristic operating-point scores. For position,

(15) $$\rho_{q}=\min\left(1,\frac{K_{p}\max(U_{q},|r_{q}\left|\right)}{18}\right),$$

which measures the residual/uncertainty magnitude in approximate PWM-equivalent coordinates. For current,

(16) $$\rho_{i}={\mathrm{clip}}_{\left[0,1\right]}\left(\frac{|{\hat{i}}_{k-1}|+U_{i,k}-I_{\mathrm{warn}}}{I_{\mathrm{soft}}-I_{\mathrm{warn}}}\right),$$

so current integrity increases when the previous corrected estimate plus uncertainty approaches the constraint band. This dependence can self-mask a sustained current under-read: the faulty measurement lowers the corrected estimate and can reduce its own relevance. It is an architectural limitation, not a proven safe decision. For position, $K_{p}\max(U_{q},|R_{q}\left|\right)/18$ omits both saturation and the speed-innovation contribution to the PD command. Away from sign changes and saturation, accepting rather than suppressing the effective position innovation changes PD demand by $\left(K_{p}K_{q}+K_{d}K_{v}/\Delta t\right)|R_{q}^{\mathrm{eff}}\left|=3.06\right|R_{q}^{\mathrm{eff}}|$ PWM in the evaluated observer. Saturation and current derating can reduce the actual change to zero. Thus, the original score is neither an exact command counterfactual nor a general bound through dead-zone sign changes. The raw impact evidence is $h_{j}=s_{j}\rho_{j}$. The recorded impact dead zone τ=0.55 gives

(17) $$g_{j}=\left\{\begin{array}{cc} 0, & h_{j}\leq \tau ,\\ \left(h_{j}-\tau \right)/\left(1-\tau \right), & h_{j}>\tau . \end{array}\right.$$

For finite measurements, CRASI uses

(18) $$w_{j,k}=\max\left(0.55,1-g_{j,k}\right),$$

whereas a missing measurement forces $w_{j,k}=0$. The 0.55 floor intentionally avoids hard switching on finite sensor values. When $g_{j}>0$, an exponential bias tracker estimates persistent finite bias (gain 0.15 for encoder residuals and 0.10 for current residuals) and subtracts it before correction. A dropout is followed by a 50-sample reacquisition interval during which the channel is re-anchored before bias tracking resumes.

The observer correction is trust-weighted:

(19) $$\begin{array}{cc} {\hat{q}}_{k} & =q_{k}^{-}+K_{q}w_{q}\left(z_{k}^{q,\mathrm{eff}}-q_{k}^{-}\right), \end{array}$$

(20) $$\begin{array}{cc} {\hat{v}}_{k} & =v_{k}^{-}+K_{v}w_{q}\left(z_{k}^{q,\mathrm{eff}}-q_{k}^{-}\right)/\Delta t, \end{array}$$

(21) $$\begin{array}{cc} {\hat{i}}_{k} & =\left(1-K_{i}w_{i}\right)i_{k}^{-}+K_{i}w_{i}z_{k}^{i,\mathrm{eff}}, \end{array}$$

with $K_{q}=0.80$, $K_{v}=0.055$, and $K_{i}=0.70$. This is the position-innovation speed correction implemented in the archived engine for the original campaign. *q* and its innovation have units of counts, *v* has counts/s, and $K_{v}$ is dimensionless; the equivalent innovation gain $K_{v}/\Delta t=5.5\;s^{-1}$. It is an alpha–beta-style model/position correction, not an independent velocity measurement. Differencing a whole-count encoder at 10 ms would amplify one-count changes to 100 counts/s; using the model prediction and a small position-innovation gain avoids introducing that raw differenced signal. No direct comparison with a separately derived velocity observation was performed, so superiority of this design is not claimed. The original update order is prediction, residual/relevance calculation using prior uncertainty/current, gate and bias update, uncertainty update, position/speed/current correction, then command calculation. The evaluated uncertainty recursions are

(22) $$\begin{array}{cc} U_{q,k+1} & ={\mathrm{clip}}_{\left[U_{q}^{0},50\right]}\;\left(0.88U_{q,k}+0.12U_{q}^{0}+0.02\left(1-w_{q,k}\right)+0.08c_{k}\right), \end{array}$$

(23) $$\begin{array}{cc} U_{i,k+1} & ={\mathrm{clip}}_{\left[U_{i}^{0},0.30\right]}\;\left(0.88U_{i,k}+0.12U_{i}^{0}+0.002\left(1-w_{i,k}\right)+0.0015c_{k}\right), \end{array}$$

where $U_{q}^{0}$ and $U_{i}^{0}$ are the calibration baselines. For uncertainty-aware methods, position uncertainty modifies the command through

(24) $$\phi_{q}\left(U\right)=\left\{\begin{array}{cc} 1, & U\leq 8,\\ \max\{0.15,(40-U)/32\}, & U>8. \end{array}\right.$$

The printed derating equation has been corrected to match the code: it reaches its 0.15 floor at U=35.2 and has no upward jump at 40. More fundamentally, from $U_{q}^{0}=0.5$, the recurrence implies $U_{q}\leq 0.5+\left(0.02+0.08\right)/0.12=1.333$ counts for any allowable trust/common evidence. Its eight-count threshold is therefore unreachable in the original campaign, including dropouts. Position-uncertainty derating is inactive and explains none of the original IAE differences. Current uncertainty remains an input to the current-envelope margin. Changing the recurrence to make position derating active would define a new controller and has not been retroactively applied to the original results.

### 2.8. Comparators, Ablations, and Post-Primary Robust-Filter Benchmark

The recorded original specification contains six methods: fixed; residual; residual + uncertainty; full CRASI; no relevance; and no common-mode. The residual threshold 0.45 maps to $|r_{j}||>2.8b_{j}$ before continuous down-weighting, with operational rejection defined as $w_{j}<0.25$. Full CRASI retains $w_{j}\geq 0.55$ for finite values and tracks bias; the primary residual methods lack both features. The archived protocol records the 0.45 value but supplies no selection log demonstrating that it is optimal, calibrated for fault detection, or selected independently of result inspection. We use it as the original simple normalized-residual reference. The no-relevance ablation retains the common-evidence rule, threshold, trust floor, and bias tracker and is the more direct component comparison. The new matched residual observer retains the original residual evidence and 0.45 threshold but adds the CRASI finite floor, bias tracker, reacquisition, and uncertainty update. It tests whether those observer features alone explain the original comparator gap.

To test whether the primary result depends on a relatively simple threshold-residual comparator, a seventh method was added *after* the original primary analysis. The adaptive Huber observer applies Huber M-estimation weights to position and current innovations, following the foundational robust-estimation principle of Huber [25] and recent Huber-based robust sensor-filtering formulations such as those from Park et al. [26]. Innovation scales are updated by an exponentially weighted, winsorized absolute residual (α=0.01, clipping at three current scales); Huber tuning is c=2.5. These scales also serve as conservative uncertainty proxies for the same command-derating envelope. The method uses neither CRASI control relevance nor common-mode subtraction. It therefore provides a stronger robust-estimation benchmark related to modern residual-based adaptive-threshold strategies for fault detection under modeling uncertainty [27]. No CRASI parameter was changed after observing this benchmark. Because it was added after inspection of the original primary results, its 420 episodes are reported as an exploratory sensitivity analysis and are excluded from the original multiplicity family.

### 2.9. Final Domains, Faults, and Paired Episode Design

The final partition contains a nominal 50% load domain and a held-out 70% load domain. The held-out domain increases speed-process noise, current noise, and encoder threshold jitter and was not used for calibration. Each domain contains seven scenarios: healthy operation; +40-count encoder bias (12–24 s); encoder drift of 3 counts/s (12–24 s); encoder dropout (12–15 s); +0.10 A current-magnitude bias (12–24 s); current gain under-read of 0.40 (12–24 s); and current dropout (12–15 s). Thirty complete 32 s episodes were run for every domain–scenario–method combination. Seeds 10,000–10,029 and three deterministic reference variants were shared across methods, yielding 2 domains × 7 scenarios × 6 methods × 30 episodes = 2520 final episodes.

This fault set intentionally includes cases in which reconfiguration should not necessarily help. A method that rejects every detectable anomaly would therefore not be considered successful unless it also improves closed-loop endpoints.

### 2.10. Endpoints and Statistical Analysis

The complete episode is the statistical unit. The original primary endpoints were (i) integral absolute tracking error $\mathrm{IAE}=\sum_{k}\left|r_{k}-q_{k}\right|\Delta t$; (ii) position-guard fraction; (iii) hard-current-violation fraction; and (iv) false-rejection fraction outside the injected fault window. Secondary endpoints included RMSE, maximum error, control total variation, soft-current-violation fraction, fault-rejection recall, detection delay where applicable, empirical uncertainty coverage, and post-fault mean absolute error.

The original primary comparison is proposed versus residual + uncertainty in each held-out sensor-fault scenario. Paired mean differences use 5000 episode-level bootstrap resamples for 95% intervals. Original two-sided paired Wilcoxon signed-rank tests use Pratt treatment of zeros and asymptotic handling of ties; six scenario tests are Holm-adjusted within each endpoint [28]. Fixed and ablation comparisons are secondary. The major-revision paired intervals also resample complete paired episodes 5000 times with a recorded seed; new comparisons are exploratory, without confirmatory *p* values or claims of familywise significance.

The adaptive-Huber benchmark and alternate-load replication were added after inspecting the primary results. They reuse the exact paired seeds and report paired effect sizes, bootstrap intervals where tabulated, nuisance rejection, and safety endpoints, but they are explicitly exploratory/sensitivity analyses: no analysis added after inspection of the original primary results is relabeled as confirmatory and no original hypothesis or threshold is changed.

### 2.11. Major-Revision Diagnostics and Exploratory Experiments

The original engine, protocol bytes, and archived metrics are preserved. A scalar-clipping instrumented extension retains update ordering and arithmetic bounds; focused checks compare all six observer variants for 2100 updates including missing measurements, saturated commands, and residual transients. All 1260 original held-out episodes are replayed and compared with their archived IAE. A further 420 paired replays quantify fault-window versus five-second recovery activity. These repeated original episodes are audits, not independent replications.

For each channel, we report the fault-window fraction with $g_{j}>0$, mean/minimum trust, rejection fraction, episodes with any action or rejection, conditional delay to first action or rejection, and recovery activity. Windows are 12≤t<24 s for finite faults and 12≤t<15 s for dropouts. The original finite-gate activity figure accidentally included t=24 s, causing the drift action at fault removal to be counted as fault-window activity; that boundary is corrected. Missing detections remain undefined for delay and are accompanied by the number detected. Zero finite rejection is guaranteed by CRASI’s trust floor and is not proof of detection or recovery.

For the immediate command diagnostic, the predicted state, bias-adjusted measurement, current/position uncertainty, and the other corrected channel are held fixed. Position acceptance uses $\left(q^{-}+K_{q}R_{q}^{\mathrm{eff}},v^{-}+K_{v}R_{q}^{\mathrm{eff}}/\Delta t\right)$ and suppression uses $\left(q^{-},v^{-}\right)$. Current acceptance uses $\left(1-K_{i}\right)i^{-}+K_{i}z_{i}^{\mathrm{eff}}$ and suppression uses $i^{-}$. Both commands pass through the exact dead-zone, saturation, and current-envelope calculation. The diagnostic $\Delta u_{j}=\left|u^{\mathrm{accept}}-u^{\mathrm{suppress}}\right|$ is a one-step quantity; it does not change uncertainty, bias, or future trajectories and is not a causal long-horizon value-of-information estimate. High position relevance is defined descriptively as $\rho_{q}\geq 0.9$, and negligible command effect as $\Delta u_{q}<1$ PWM.

The matched residual observer adds CRASI’s finite trust floor and bias tracker to the original raw-anomaly/0.45 residual rule, retaining the same prediction, correction gains, reacquisition, uncertainty, and envelope. It uses all 30 original paired seeds in all seven held-out scenarios (210 new episodes). The existing no-relevance ablation provides the narrower comparison holding common subtraction and the 0.55 action threshold fixed.

New simultaneous-fault tests use seeds 20000–20029 and the same three reference variants. Encoder bias (+40 counts) or drift (3 counts/s) is paired with current gain 0.40 over the same [12,24) s window. Four methods (CRASI, no common-mode, matched residual, fixed) yield 240 episodes. When $a_{q}=a_{i}=1$, $s_{q}=s_{i}=0.5$ and $s_{j}\rho_{j}\leq 0.5<0.55$; both finite gates are necessarily zero. We report the frequency of this saturated-anomaly state and its conditional no-action fraction, rather than labeling every non-action sample a classification error.

Current-relevance sensitivity uses seeds 21000–21029. Besides the original corrected-estimate relevance, the model-only alternative replaces $|{\hat{i}}_{k-1}|$ with $|i_{k}^{-}|$, and the conservative alternative uses $\max(|{\hat{i}}_{k-1}|,|i_{k}^{-}|)$. All other equations, including the current safety envelope, remain unchanged. Model prediction is independent of the contemporaneous faulty current reading but depends on past control and assumed plant validity; neither alternative is an independent truth measurement. Four methods including fixed give 120 episodes. An exploratory masking timeline also shows true/estimated current, relevance, gate, and actual command.

The one-factor parameter sweep uses separate seeds 23000–23029, all three references, and encoder bias, encoder drift, and current under-read. It tests the original setting plus β∈{0,0.25,0.75,1}, τ∈{0.35,0.45,0.65,0.75}, PWM divisor D∈{6,12,24,36}, and finite trust floor f∈{0,0.25,0.75,1}, changing one factor at a time. The original settings (0.5,0.55,18,0.55) remain fixed in the central run. Fixed, residual + uncertainty, and matched residual share those same new seeds, totaling 1800 episodes. This is a local sensitivity study of three selected faults, without joint-factor coverage or parameter optimization.

Guard stress uses seeds 22000–22029, reference amplitude multiplied by 1.4, and true-plant current-map multipliers 2 and 3. All commanded reference positions remain within the 260-count guard (maximum 252 counts). These current multipliers are synthetic assumptions; speed dynamics, noise, original controller parameters, observer map, and guards remain fixed. Current under-read and paired encoder-bias/current-under-read are tested with CRASI, model-only/conservative relevance, fixed, and residual + uncertainty; encoder bias is additionally tested with CRASI and fixed (720 episodes). Worst-case absolute true current, soft/hard-current fractions, maximum position, and guard fractions are reported. The surrogate’s ±300-count contact boundary is retained and is a structural limitation of extreme-position stress. These stress runs challenge safety interpretation and are not physical motor validation.

### 2.12. Embedded-Oriented Reference Implementation and AVR Static Audit

The package includes a C99 integer integrity kernel in which normalized anomaly, relevance, gate, trust and derating factors are represented in Q15; position residual/uncertainty use centi-count units and current quantities use microamperes. A freestanding AVR-target variant was compiled for ATmega2560 with Clang 17.0.0 using -target avr -mmcu=atmega2560 -Os -ffreestanding -fno-builtin -nostdlib. The relocatable standalone kernel contains 1750 bytes of .text, no .data/.bss, a 12-byte persistent state object and 22-byte diagnostic object under the AVR ABI. Compiler stack-usage analysis reports 45 bytes for crasi_integrity_step and zero for reset. The kernel contains no dynamic allocation or floating-point operations.

These measurements establish static resource plausibility, not full-firmware memory use or real-time execution. They exclude UART, ISR, sensing, motor-drive and runtime/link support, and no target-cycle/WCET measurement was possible in this environment. Therefore, this paper does not claim that the complete CRASI firmware has been timed at 100 Hz on ATmega2560 hardware.

### 2.13. Revision-Era Direct Proteus Audit Status

A revision-era 18-cell direct Proteus encoder-bias audit was attempted with frozen controller parameters. The original strict acquisition gates accepted only 10 of 18 cells, and the complete 18-cell analysis required a disclosed amendment after partial performance inspection. The audit is therefore not a fresh confirmatory validation and is not used to support the main results or conclusions. Its allocation, original protocol, amendment, per-cell summary metrics, hashes, and executable acquisition/analysis code are retained by the authors for transparency and are available upon reasonable request. A separate fresh-validation protocol with fixed acquisition and analysis criteria has also been retained by the authors; it contains no outcome data and must be executed on new runs before any confirmatory direct-simulation claim.

OpenAI Codex (GPT-6, accessed October 2026) assisted revision software implementation and manuscript editing. All numerical results reported in the manuscript are computed from archived source records and executable analysis code and were checked by the authors.

## 3. Results

The original archive contains 84 expected domain–scenario–method groups and 2520 complete episodes with no duplicate keys or missing primary outcomes. Its protocol bytes match the supplied digest, establishing file integrity rather than independently verified chronology. Existing exploratory studies add 420 Huber and 840 alternate-load episodes. The revision reruns all 1260 original held-out episodes for instrumentation and compares their IAE with the archived metrics; the maximum discrepancy is below $10^{-7}$ count·s. These are audit repeats, not new independent evidence. Additional unique revision episodes and their seeds are recorded in the machine-readable revision audit retained by the authors and are available upon reasonable request.

### 3.1. Healthy Operation Is Not Sacrificed

In the nominal domain, mean healthy IAE was 730.436 count · s for fixed control and was numerically identical for the proposed method; the other methods also differed by less than $2\times 10^{-6}$ count · s. In the held-out domain, fixed and proposed control were again identical at 903.160 count · s. Residual+uncertainty increased healthy IAE by only 0.043 count · s, while the two ablations differed from fixed by at most 0.101 count · s. Thus, the proposed method’s improvements in faulted cases are not purchased by a healthy tracking penalty.

Healthy empirical uncertainty coverage was 1.000/1.000 (position/current) in the nominal final domain and 1.000/0.987 in held-out operation for the proposed method. Because the held-out plant is outside the calibration load range and the uncertainty recursion is adaptive, these numbers are descriptive; no formal held-out coverage guarantee is asserted.

### 3.2. Primary Held-Out Sensor-Fault Results

**Figure 3:**
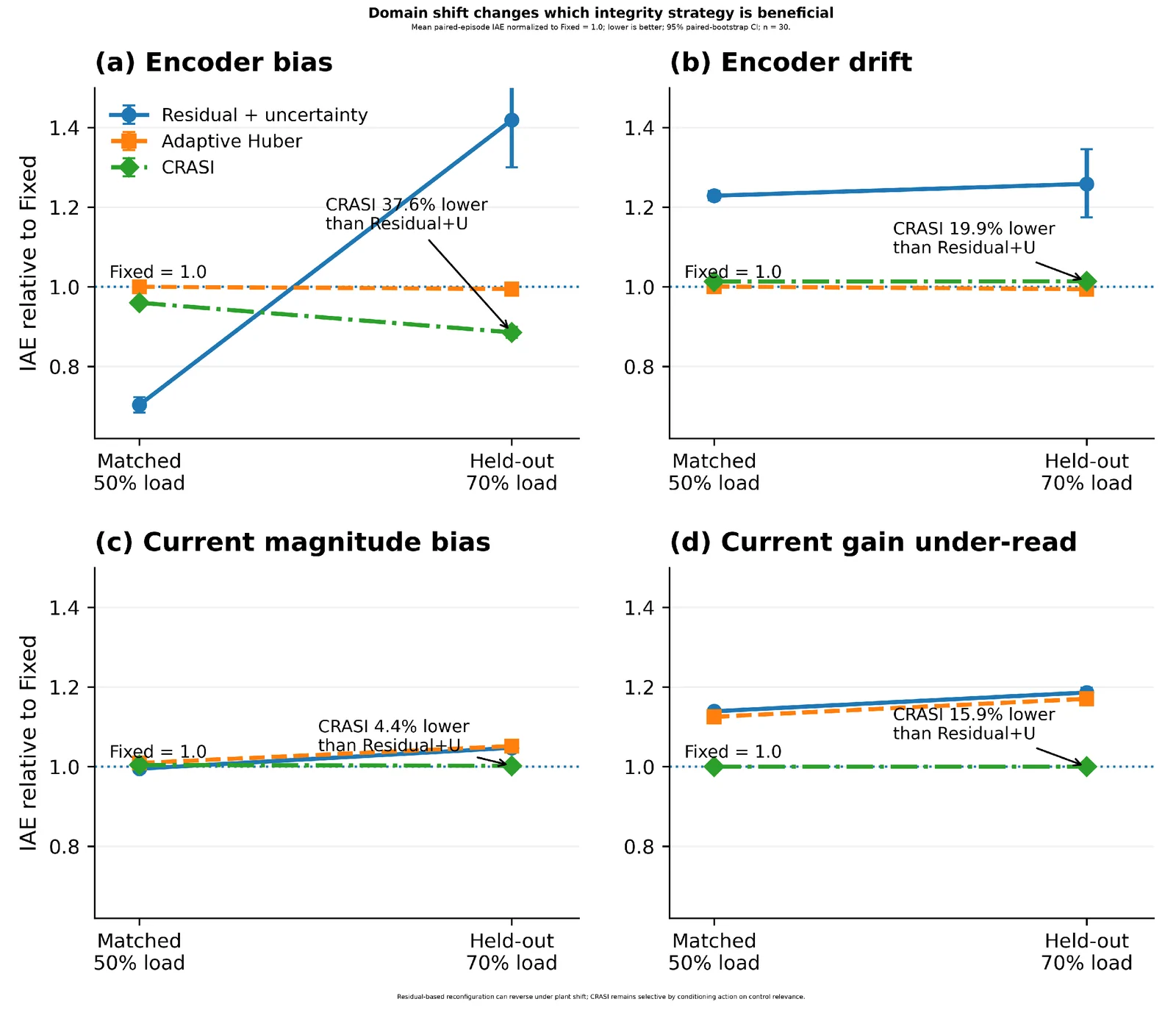
Domain-shift crossover in closed-loop tracking performance. (**a**) Encoder bias; (**b**) encoder drift; (**c**) current magnitude bias; and (**d**) current gain under-read. Mean episode-level integral absolute error (IAE) is normalized within each domain to the fixed-controller baseline (=1); lower values indicate better tracking. Points show means over 30 paired episodes and error bars denote 95% paired-bootstrap confidence intervals. The encoder-bias case makes the crossover explicit: residual-plus-uncertainty reconfiguration is beneficial in the matched 50% load domain but becomes strongly detrimental in the held-out 70% load domain, whereas CRASI remains below the fixed baseline. The same figure reports encoder drift, current magnitude bias, and current gain under-read and includes adaptive Huber as the post-primary exploratory robust-estimation comparator. Held-out CRASI reductions relative to residual-plus-uncertainty are 37.6%, 19.9%, 4.4%, and 15.9%, respectively.

Figure 3 and Table 1 summarize the held-out 70% load results and place them against the matched 50% load domain. Relative to residual + uncertainty control, the proposed method reduced mean IAE by 37.6% for encoder bias, 19.9% for encoder drift, 4.4% for current magnitude bias, and 15.9% for current gain under-read. The paired confidence intervals excluded zero and the Holm-adjusted tests remained significant in these four scenarios. Encoder and current dropouts were statistical ties.

**Figure 4:**
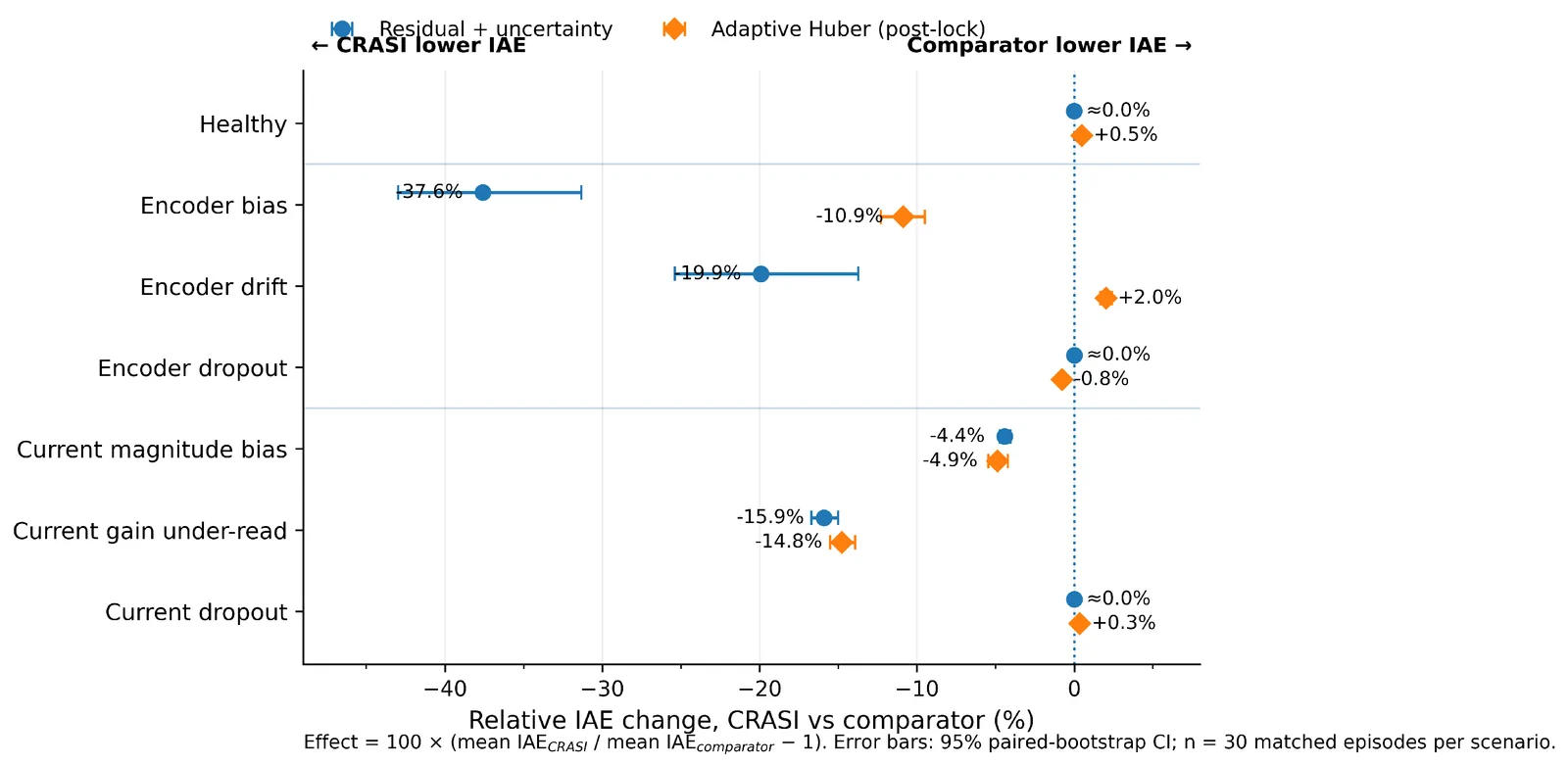
Paired effect estimates for CRASI in the held-out 70% load domain. Effects are expressed as $100({\bar{\mathrm{IAE}}}_{\mathrm{CRASI}}/{\bar{\mathrm{IAE}}}_{\mathrm{comparator}}-1)$, so negative values indicate lower tracking error for CRASI. The left-pointing arrow denotes the direction in which CRASI has lower IAE, whereas the right-pointing arrow denotes the direction in which the comparator has lower IAE. Points denote paired-sample effect estimates and horizontal bars denote 95% paired-bootstrap confidence intervals across 30 matched episodes per scenario. All seven held-out scenarios are shown, including healthy operation and both dropout cases. Residual-plus-uncertainty is the original primary comparator; adaptive Huber is the post-primary exploratory robust-estimation comparator. The figure therefore exposes both the large finite-fault gains and the small adverse/null cases rather than selecting only favorable conditions.

The paired effects are shown directly in Figure 4. The asymmetry is important: large finite biases and gain faults can create sustained but misleading residual evidence under plant shift, whereas a dropout is unambiguous to every method and therefore offers little scope for relevance-aware improvement.

### 3.3. Nuisance Reconfiguration Under Plant Shift

For held-out encoder bias, residual + uncertainty rejected a sensor outside the injected fault window for 22.65% of eligible samples on average; the proposed method’s false-rejection fraction was 0. The paired difference was −0.2265 [95% CI −0.2890,−0.1638], Holm-adjusted $p=7\times 10^{-6}$. For encoder drift, the corresponding values were 25.02% versus 0, difference −0.2502 [−0.3118,−0.1874], and Holm-adjusted $p=8\times 10^{-6}$. No other held-out scenario produced false rejection for either of these two methods.

The domain-shift interaction is especially visible for encoder bias. In the nominal final domain, residual + uncertainty achieved 744.6 count · s IAE, outperforming fixed control (1057.3) and the proposed method (1015.5). In the held-out domain, however, the same residual strategy deteriorated to 1694.2, while proposed control was 1057.3 and fixed control 1192.5. This crossover is the central result: the proposed method sacrifices some aggressiveness when the residual is trustworthy in exchange for substantially greater robustness when residual magnitude is contaminated by plant shift.

**Figure 5:**
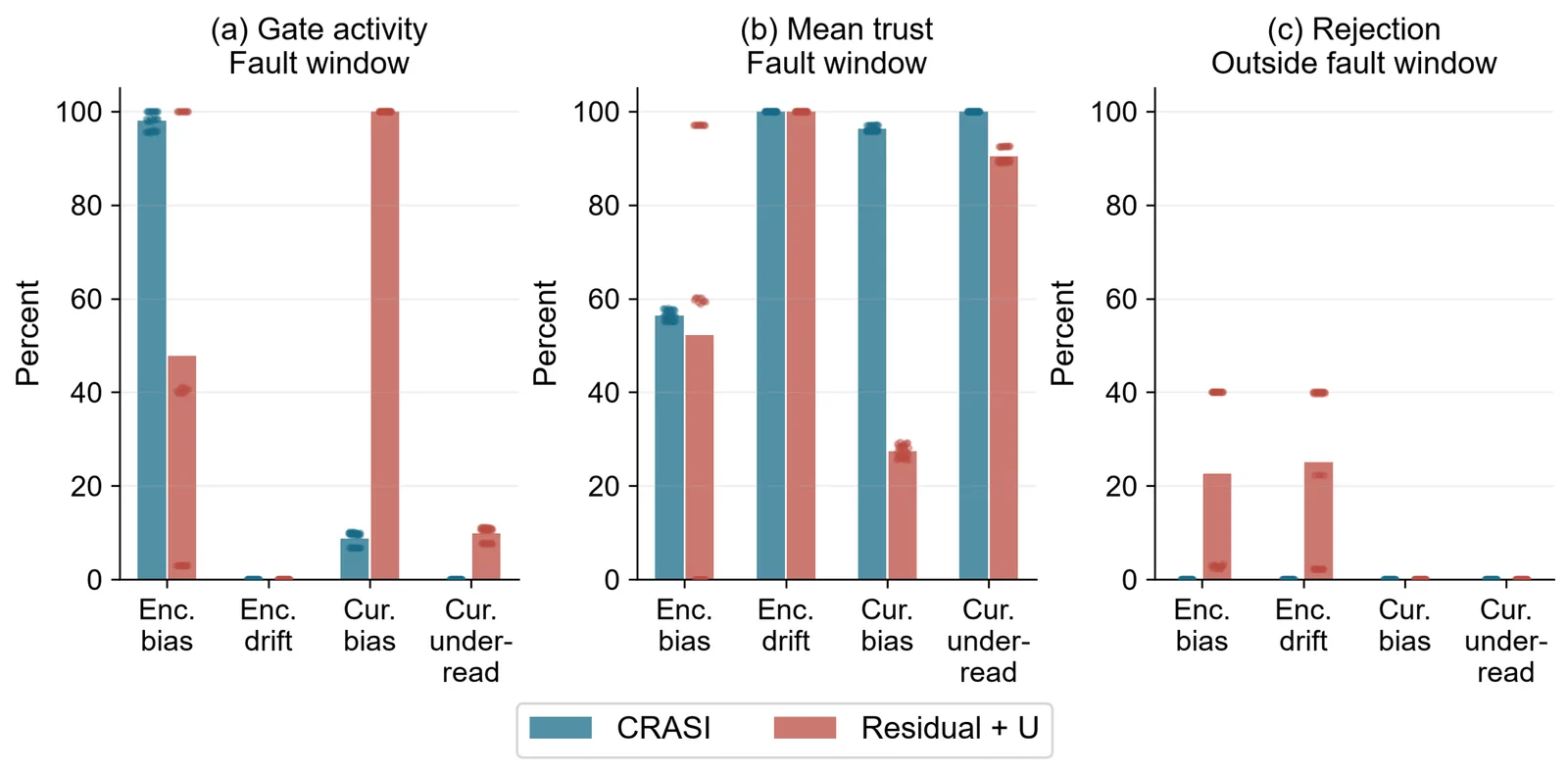
All-episode finite-gate activity and trust in the held-out domain. (**a**) Mean fault-window gate activity; (**b**) mean fault-window trust; and (**c**) operational rejection outside the fault window. Episode summaries use every fault-window sample from all 30 seeds. Bars show mean fractions with nonzero encoder/current gates and mean trust; dropout missing samples are handled separately. Residual-plus-uncertainty operational rejection is also shown. This replaces representative-only gate occupancy as the mechanism summary; complete per-episode diagnostics are supplied.

Figure 5 summarizes gate occupancy over all 30 held-out episodes per condition. Representative phase-space occupancy alone cannot explain a paired tracking difference. Finite action ($g_{j}>0$), trust reduction, bias tracking, and operational rejection ($w_{j}<0.25$) are distinct: finite CRASI values cannot meet the rejection definition because of the 0.55 floor. Rejection recall can therefore be zero even while bias accommodation is active.

### 3.4. Representative Mechanism Timeline

**Figure 6:**
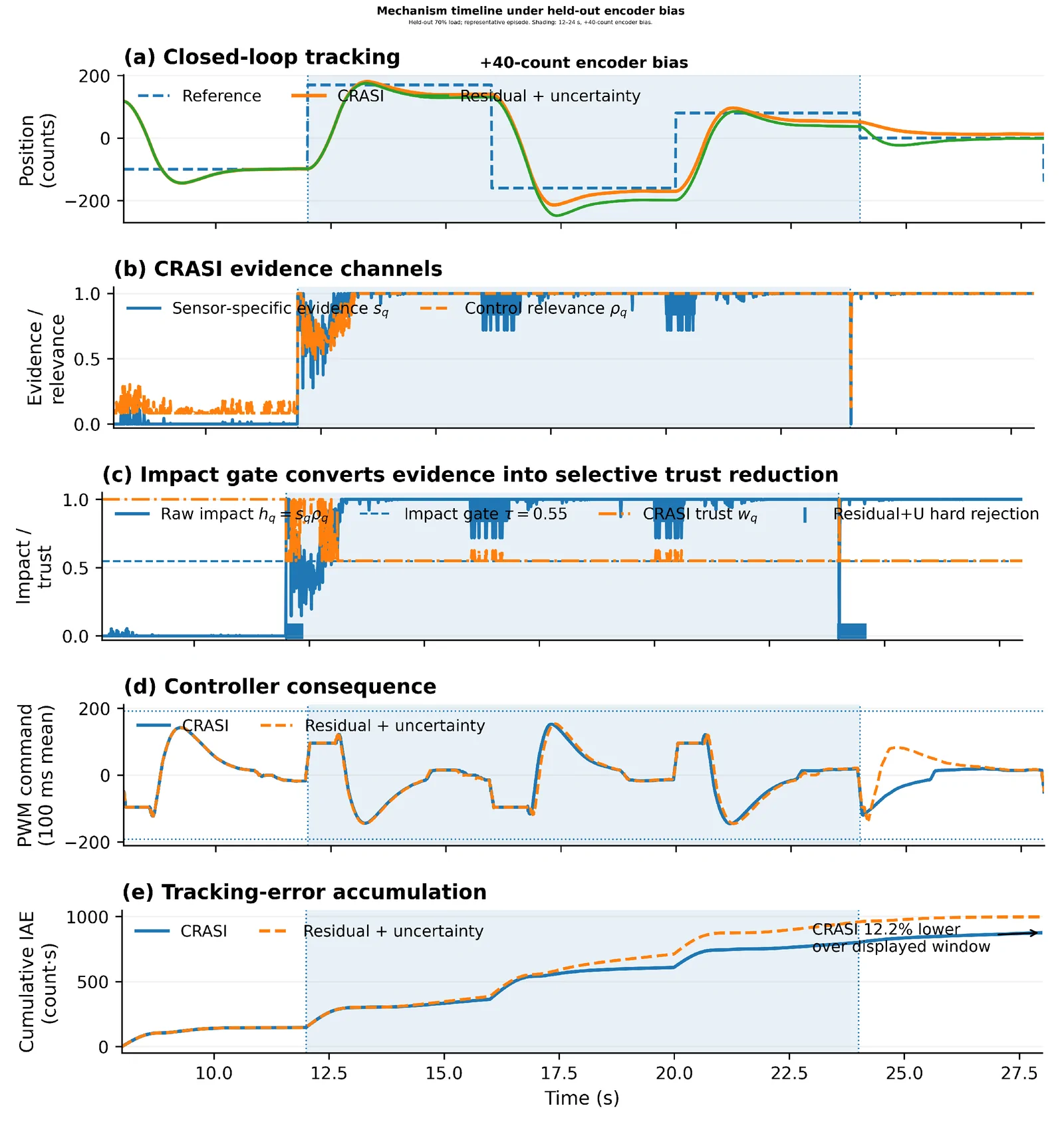
Mechanistic timeline for a representative held-out encoder-bias episode. The 70% load episode is shown from 8–28 s, with the shaded 12–24 s interval indicating the injected +40-count encoder bias. (**a**) CRASI and residual-plus-uncertainty tracking responses relative to the common position reference. (**b**) CRASI sensor-specific anomaly evidence $s_{q}$ and control relevance $\rho_{q}$. (**c**) Their product $h_{q}=s_{q}\rho_{q}$, the originally specified impact threshold τ=0.55, resulting finite-sensor trust $w_{q}$, and residual-plus-uncertainty hard-rejection events. (**d**) Controller commands shown as 100 ms moving averages for visual clarity; the underlying analysis uses the raw 100 Hz commands. (**e**) Cumulative absolute tracking error over the displayed interval; the rightward annotation arrow identifies the 12.2% lower cumulative IAE for CRASI over the displayed window. CRASI accumulates 12.2% less IAE than residual-plus-uncertainty in this representative window; this trace-level quantity is illustrative and distinct from the paired scenario mean.

Figure 6 synchronizes the causal chain for the representative held-out encoder-bias episode. The plot is not used for inference; it is a trace-level explanation of how the saved diagnostics map the injected bias to evidence, relevance, trust, command behavior, and the resulting tracking-error accumulation. Over the displayed 8–28 s window, CRASI accumulates 12.2% less IAE than residual-plus-uncertainty; the study-level paired mean effect remains the 37.6% value reported above.

### 3.5. Adverse and Null Results

The proposed method was not universally best. Held-out fixed control slightly outperformed CRASI for encoder drift and current magnitude bias; current gain under-read was exactly equal to fixed control. Both dropout comparisons were effectively null. Table 2 reports paired intervals against fixed, matched residual, and Huber rather than interpreting every gap against residual + uncertainty as successful fault accommodation.

Removing common-mode subtraction changed original held-out IAE only slightly for encoder bias and drift and slightly improved current under-read. Removing relevance increased under-read IAE from 794.5 to 934.1 count·s but had little independent effect on encoder drift. This supports a scenario-specific effect of inhibiting integrity action; it does not establish relevance as the sole mechanism behind all improvements. The new matched residual comparison tests the combined trust-floor/bias-tracker contribution, and paired multi-sensor tests expose a limitation of common subtraction.

### 3.6. Post-Primary Exploratory Adaptive Huber Robust-Observer Benchmark

**Figure 7:**
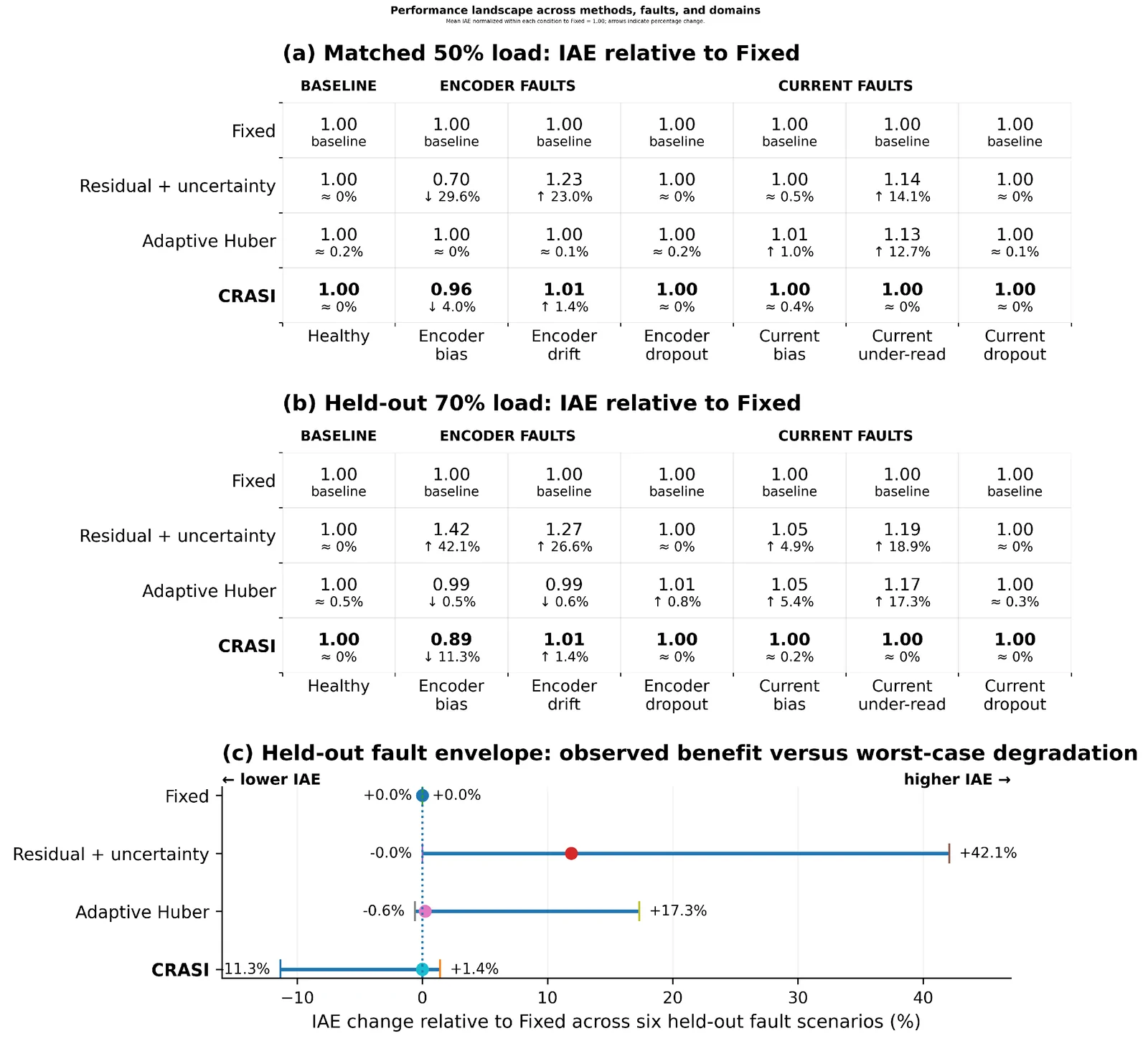
Performance landscape across control strategies, sensor-degradation modes, and plant domains. Panels (**a**,**b**) report mean episode-level IAE normalized within each condition to the fixed-controller baseline (=1.00); values below unity indicate improved tracking and values above unity indicate degradation. Panel (**a**) shows the matched 50% load domain and panel (**b**) the held-out 70% load domain. The adaptive-Huber row is a post-primary exploratory sensitivity benchmark. Panel (**c**) summarizes the observed range of mean IAE changes relative to fixed control across the six held-out fault scenarios: CRASI spans an 11.3% improvement to a 1.4% degradation, residual-plus-uncertainty spans approximately 0% to 42.1% degradation, and adaptive Huber spans approximately 0.6% improvement to 17.3% degradation. In panel (**c**), the blue horizontal segment represents each method’s observed best-to-worst range, the colored endpoint markers identify the two extremes, and the dotted blue vertical line marks zero change. The panel (**c**) envelope is descriptive across fault scenarios and is not a confidence interval.

The existing post-analysis Huber benchmark shows selective differences (Figure 4 and Figure 7), with a separate paired held-out summary in Table 2. CRASI reduced mean IAE relative to Huber for encoder bias, current bias, and under-read but lost 2.0% for drift, 0.47% for healthy operation, and 0.34% for current dropout. Huber finite-encoder nuisance rejection was about 0.47–0.52%, below the original residual comparator’s 22.6–25.0%. Huber was added after original result inspection and remains exploratory.

Figure 7 consolidates the method–fault landscape across both final domains. It also makes the robustness trade-off visible without converting it into a universal-ranking claim: across the six held-out fault scenarios, CRASI ranges from an 11.3% improvement to a 1.4% degradation relative to fixed control, whereas residual-plus-uncertainty ranges from essentially no benefit to 42.1% degradation and adaptive Huber from about 0.6% improvement to 17.3% degradation.

### 3.7. Surrogate Consistency and Alternate-Load Sensitivity Analysis

The retrospective lower-load cross-check showed decreasing speed error as the measured load approached the 50% lookup condition: speed sMAPE was 13.4, 10.9 and 8.5% at 10, 20 and 30% load, with current MAE 3.10–5.92 mA. This does not validate 70% operation, but it provides an auditable lower-load consistency check of the surrogate.

**Figure 8:**
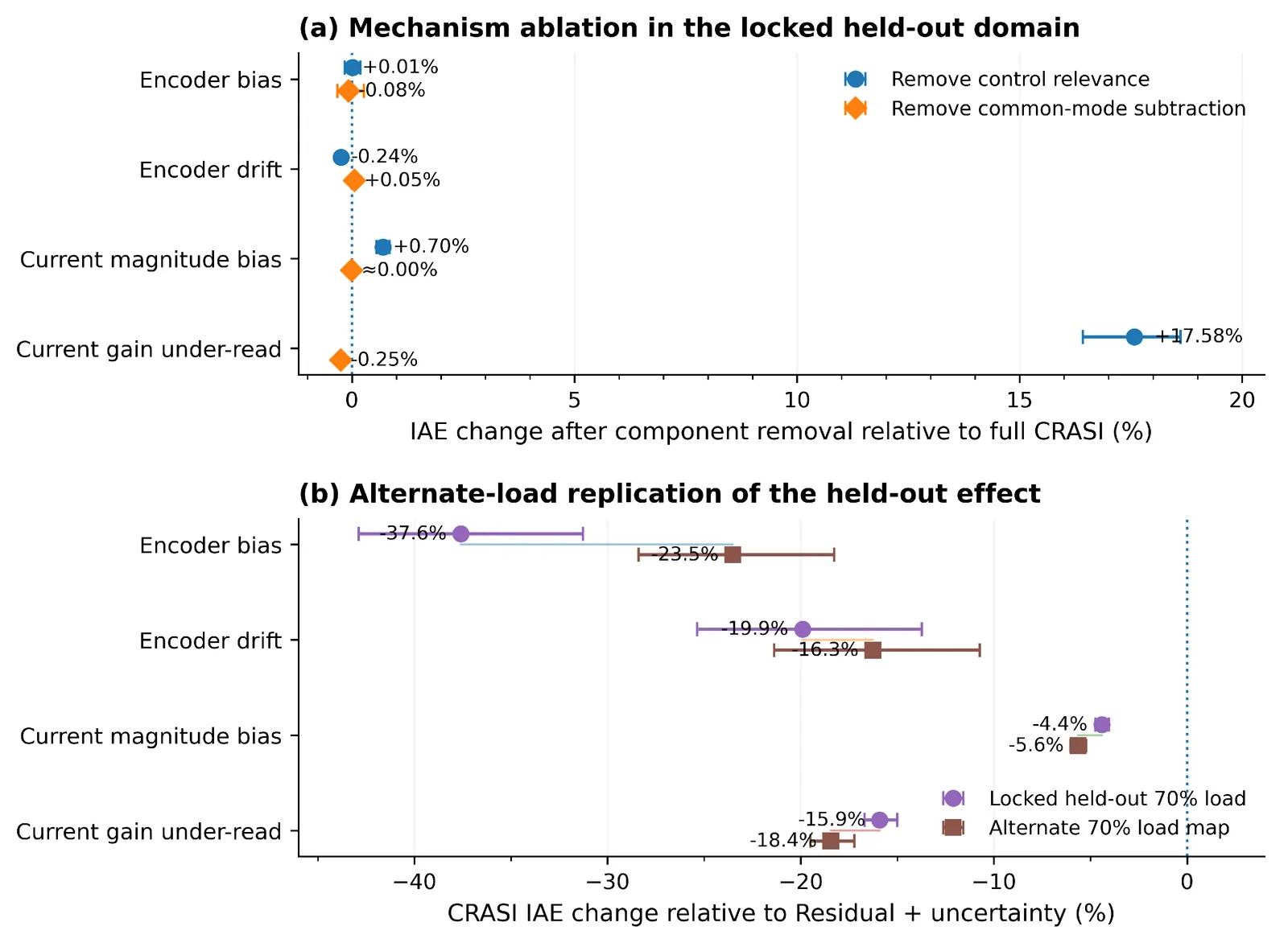
Mechanism ablation and alternate-load robustness. (**a**) Paired ablation effects in the original held-out 70% load domain, expressed as the percentage change in mean IAE after removing one CRASI component relative to the complete method; positive values therefore indicate worse tracking after removal. Removing control relevance has its largest consequence for current gain under-read (about +17.6%), whereas removal of common-mode subtraction produces comparatively small changes across the evaluated conditions; the encoder-drift ablation is a small adverse/null result rather than evidence of universal benefit. (**b**) Post-primary exploratory sensitivity analysis of the CRASI-versus-residual-plus-uncertainty effect under an independently constructed alternate 70% load map. Negative values indicate lower IAE for CRASI. The effect direction is preserved for encoder bias, encoder drift, current magnitude bias, and current gain under-read. Points are paired mean-ratio effects and horizontal bars are 95% paired-bootstrap confidence intervals over 30 matched episodes per condition.

More importantly, Figure 8 summarizes both the mechanism ablation (panel a) and the alternate-load sensitivity analysis (panel b). The alternate-load sensitivity analysis added after the primary results changed the 70% load construction itself. Under the per-PWM load-trend extrapolation, CRASI again reduced IAE relative to residual + uncertainty by 23.5% for encoder bias, 16.3% for encoder drift, 5.65% for current magnitude bias and 18.4% for current gain under-read (Figure 8b). Nuisance rejection for residual + uncertainty remained 17.1% and 21.7% in the two finite encoder cases, versus 0 for CRASI. Compared with adaptive Huber on this alternate plant, CRASI was 5.7% better for encoder bias, 4.1% for current bias and 16.5% for current under-read, whereas Huber was better for healthy operation, encoder drift, and the dropout cases. Four of 30 Huber encoder-bias episodes briefly crossed the ±260-count position guard (maximum episode guard fraction 0.1875%); CRASI, fixed, and residual + uncertainty had no guard crossings in the alternate-load sensitivity analysis. No method produced a hard-current violation.

### 3.8. AVR-Target Static Resource Audit

The standalone integer CRASI integrity kernel compiled for the ATmega2560 AVR target to 1750 bytes of code. Target-ABI persistent state and diagnostics occupy 12 and 22 bytes, and compiler stack-usage analysis reports 45 bytes for the integrity step. No floating point or dynamic allocation is present. These measurements materially strengthen embedded feasibility relative to a host-only C smoke test, but no execution-time claim is made because the kernel was not linked into the complete firmware or cycle-profiled on the target.

### 3.9. Safety Endpoints and Uncertainty Behavior

Across all 2520 original episodes, every method had zero hard-current and position-guard violations. The nominal domain had zero soft-current violations; held-out means ranged from 5.49% to 5.56%, with CRASI at 5.50%. These near-equal outcomes provide no evidence of safety superiority. The held-out worst current and the adverse guard-stress results are reported below; zero crossings in the lower-stress original design do not establish a protective guarantee.

Faulted-channel uncertainty coverage deteriorated for finite encoder bias/drift and current faults. These values do not demonstrate fault-conditional calibration. Position uncertainty is an adaptive diagnostic scale in this campaign and never activates its command envelope; current uncertainty changes its margin but no independently validated safety benefit follows.

### 3.10. Revision Audit: Mechanism, Detection, and Recovery

The instrumented rerun exactly reproduced all 1260 held-out archived IAE values (maximum absolute discrepancy 0.0 count·s). Observed $U_{q}$ never exceeded 0.8436 counts; $\phi_{q}=1$ at every audited sample for every method. These observations agree with the analytic 1.333-count upper bound. Table 3 reports the finite-fault channels over all 30 episodes per condition.

Encoder-bias CRASI action occurs in 97.97% of fault samples and supports active accommodation. Encoder drift and current under-read have zero CRASI gate activity in every fault window. For drift, CRASI and residual + uncertainty fault-window MAE are nearly identical (52.3773 and 52.3792 counts), whereas five-second recovery MAE is 30.46 versus 59.42. CRASI encoder action occupies 93.63% of recovery samples with mean trust 0.5958. The original 19.9% episode-level difference therefore largely reflects post-removal recovery/comparator behavior rather than drift accommodation during the injected window. Under-read CRASI uses unit current trust throughout the fault and equals fixed control; its IAE gap against residual + uncertainty is avoidance of comparator-induced degradation, with self-masking still present.

Operational rejection endpoints were evaluated for every original domain–fault–method group. CRASI rejection recall is zero for all finite faults because the finite trust floor prevents rejection. All 30 episodes of each dropout detect the missing channel at onset, with 100% rejection recall and zero delay. Finite-action delay was evaluated separately; an undetected event is represented as missing delay with its detection count, not assigned a zero delay. Tracking and recovery remain independent endpoints.

### 3.11. Immediate Command Diagnostic and Matched Comparators

**Figure 9:**
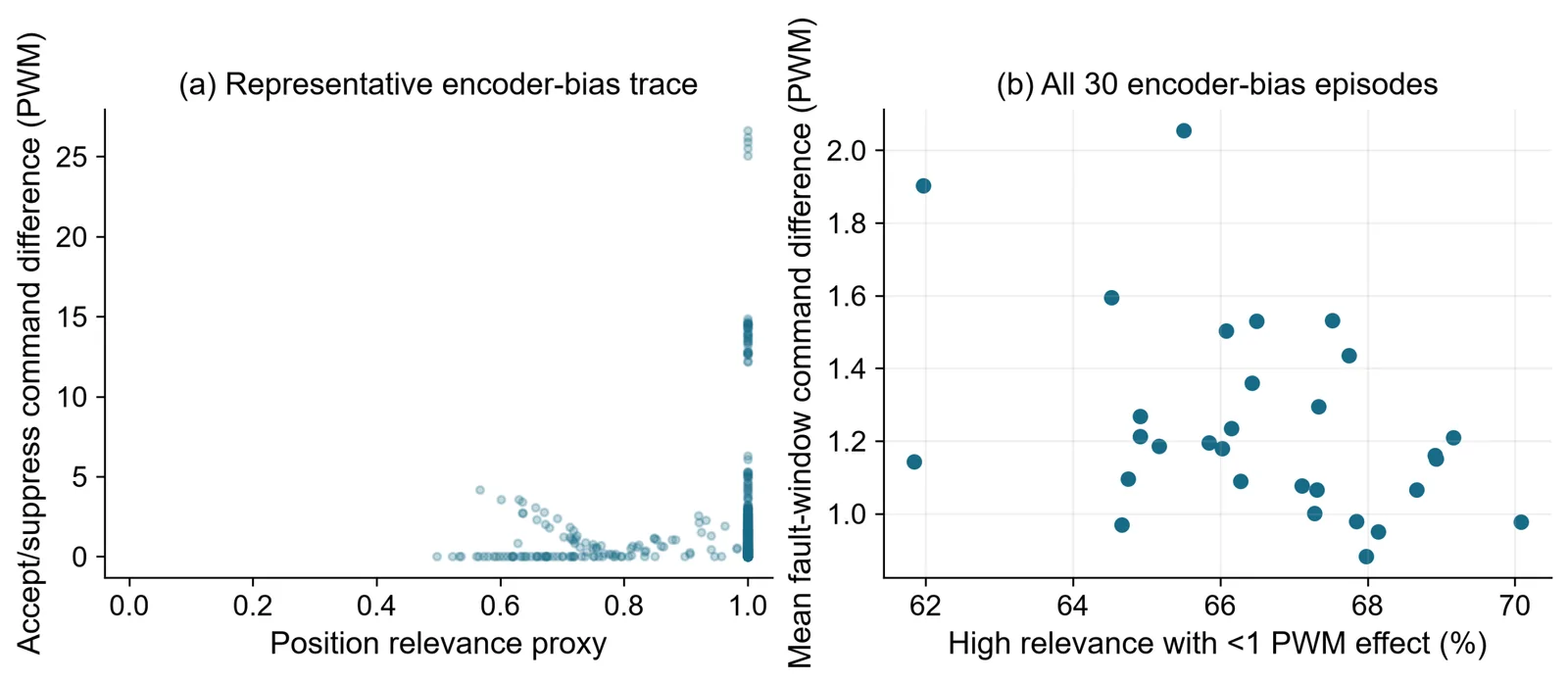
Immediate accept/suppress command diagnostic. (**a**) Effective position-innovation acceptance versus suppression for seed 10000 during the correct [12,24) s encoder-bias window; light open circles denote individual fault-window samples. (**b**) Episode-level summaries for all 30 held-out encoder-bias seeds; filled dark circles denote one summary point per episode. The common blue-green color identifies the same command-difference diagnostic in both panels, while marker fill distinguishes sample-level from episode-level summaries. Prediction, bias state, uncertainty, and the other channel are held fixed; the diagnostic is not a future-trajectory intervention.

In encoder-bias CRASI episodes, high position relevance occurs for 95.41% of fault samples. Conditional on high relevance, the immediate accept/suppress difference is below one PWM for 66.52% and exactly zero for 14.45% (means of episode fractions). Mean fault-window $\Delta u_{q}$ is 1.243 PWM and mean within-episode Spearman correlation between $\rho_{q}$ and $\Delta u_{q}$ is 0.065. This confirms that the relevance proxy is a weak surrogate for immediate command impact in these biased, saturated, bias-corrected trajectories (Figure 9). It is not relabeled as an exact counterfactual metric.

The matched residual observer reduces the encoder-bias gap from 636.93 to 11.15 count·s (37.6% to 1.04%); it is 1.09% better than CRASI for drift. Thus, the finite floor and bias tracker account for much of the original bias/drift comparator gap. CRASI remains 14.95% better than matched residual for under-read while exactly matching fixed control. Against fixed, the adverse drift and current-bias differences have intervals above zero, whereas both dropout differences and under-read are exactly zero. These results restrict the claimed mechanism and show selective performance rather than universal fault correction.

### 3.12. Simultaneous Faults and Current Self-Masking

In the paired encoder-bias/current-under-read study, both anomalies saturate in 5.58% of fault-window samples on average and occur in all 30 episodes. Every such sample has both finite gates equal to zero, as predicted algebraically. Encoder action occurs in 91.15% of all fault samples; current action remains zero. Removing common subtraction increases encoder action to 98.31% and reduces mean IAE from 1048.27 to 965.70 count·s. Paired drift/under-read produces no fault-window action in CRASI and is worse than fixed control. The common subtraction is therefore vulnerable to correlated anomalies; the original single-fault test did not reveal this boundary.

**Figure 10:**
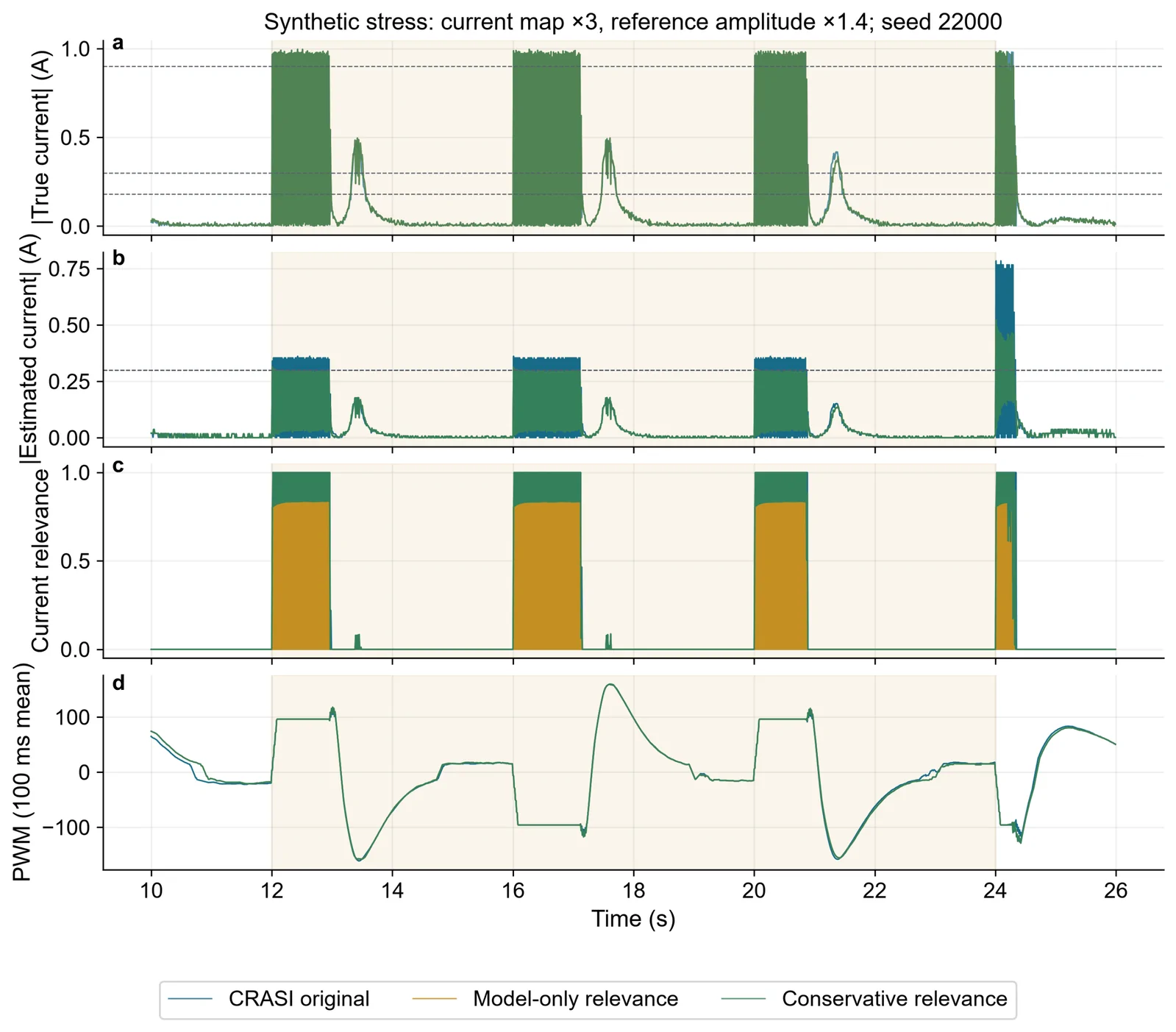
Current under-read and controller behavior on a synthetic guard-stress trajectory (seed 22000, current map multiplier 3, reference multiplier 1.4). From top to bottom, (**a**) true current, (**b**) corrected/estimated current, (**c**) current relevance, and (**d**) actual PWM command are shown. Blue denotes CRASI original relevance, amber denotes model-only relevance, and green denotes conservative relevance; where curves coincide, the trajectories are nearly identical rather than missing. Shading is the fault window. Dashed true-current lines mark warning (0.18 A), soft (0.30 A), and hard (0.90 A) levels. Only PWM is smoothed over 100 ms for display; metrics use raw 100-Hz samples.

On separate under-read seeds, the original current relevance is zero for 71.04% of fault samples with true current above the 0.30-A soft limit (mean conditional episode fractions). Model-only and conservative relevance remove this zero-relevance masking in the baseline surrogate and activate current integrity in 9.17% of fault samples but increase IAE from 795.71 to 934.61 count·s. They alter the relevance score alone and do not create a sensor-independent safety envelope. Their model also underestimates current under the synthetic stress multipliers; Figure 10 shows that independent-of-reading relevance is not equivalent to independent current protection.

### 3.13. Separate-Seed Parameter Sensitivity

Table 4 summarizes the range and least favorable setting observed in the one-factor sensitivity study.

For encoder bias, all tested one-factor intervals versus residual + uncertainty remain below zero. For encoder drift, all tested one-factor intervals versus residual + uncertainty remain below zero. For current gain underread, all tested one-factor intervals versus residual + uncertainty remain below zero. The full paired analysis against residual + uncertainty, matched residual, and fixed control is retained in the study analysis record and is available from the corresponding authors upon reasonable request. A retained advantage against the original comparator does not imply improvement over fixed or matched control; no configuration is selected using these exploratory outcomes.

The stronger comparisons identify loss of advantage within the tested range. For encoder bias, the matched-residual interval includes zero at β=1, τ=0.75, and trust floors 0.75 and 1; with floor zero, CRASI is worse by 14.21% [7.13, 21.33]. Against fixed at floor zero the mean change is +4.08% [−4.32, 12.99], so no resolved advantage remains. Under-read is exactly null against fixed at every setting. Drift is adverse against fixed/matched at the original setting. These comparator-dependent boundaries are more informative than the retained gap against the aggressive original baseline.

**Figure 11:**
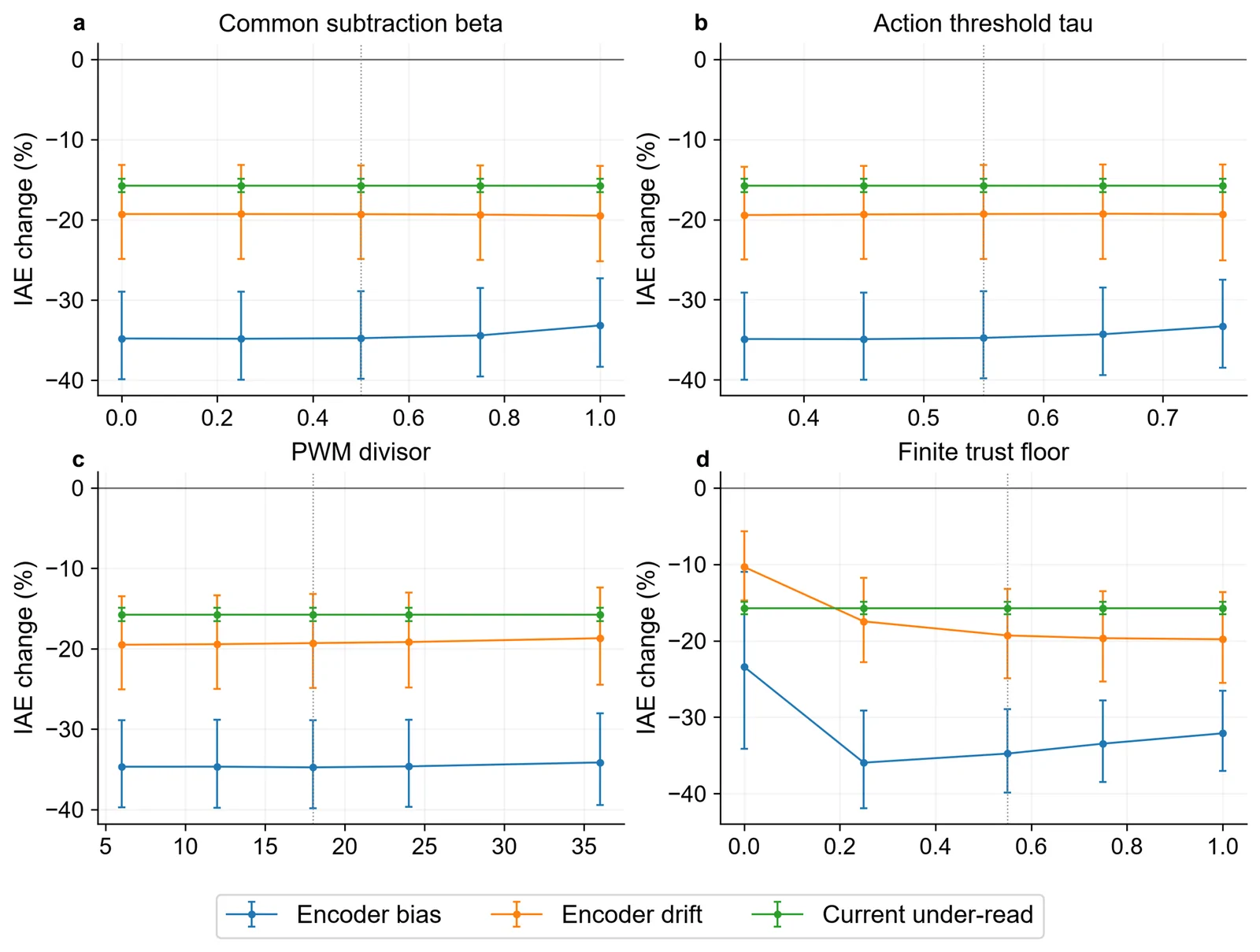
One-factor sensitivity on separate seeds 23000–23029 (n=30 per condition). (**a**) Common-subtraction factor β; (**b**) action threshold τ; (**c**) PWM divisor *D*; and (**d**) finite trust floor *f*. Lines show paired mean-ratio IAE changes versus residual + uncertainty; bars are 95% episode-bootstrap intervals. Dotted vertical lines mark original parameter values. All other parameters are fixed. Joint parameter interactions and untested fault severities remain outside this sweep.

Figure 11 shows the corresponding one-factor sensitivity profiles for the four varied parameters.

### 3.14. Guard-Stress Outcomes and Safety Limits

Table 5 summarizes the synthetic current-under-read guard-stress outcomes for the tested current-map multipliers.

The original held-out audit peaks at 0.3531 A and 259.01 counts, explaining why the 0.90-A guard is not challenged there. In the synthetic stress study, worst true current reaches 1.0041 A. Hard-current and position-guard crossings occur despite the unchanged base envelope. The largest complete-episode current peaks are shared pre-fault transients, so they do not support a ranking of fault accommodation; fault-window masking and full-episode violation fractions provide complementary information. Model-based relevance improves some conditional activity/violation fractions but does not eliminate violations or recover an independent current truth. Tracking improvements under these stresses cannot establish safety superiority. Independent measured transients and a separately validated protective envelope remain required.

### 3.15. Exploratory Direct-Audit Assessment and Protection Trips

The revision-era direct Proteus audit is retained only as a transparency record and is not used as validation. The original strict acquisition gates accepted 10 of 18 cells; the complete 18-cell analysis required a disclosed amendment after partial outcome inspection. Protection interventions also materially affect interpretation at the 50% setting: trips occurred in 2/3 fixed-control profiles, 1/3 residual-plus-uncertainty profiles, and 1/3 CRASI profiles. Descriptively, the mean IAE over trip-free cells was 1492.46 count·s for fixed (n=1), 1995.30 for residual-plus-uncertainty (n=2), and 1457.36 for CRASI (n=2). Only reference profile C2 was trip-free for all three methods at 50%, where IAE was 1492.46, 1756.55, and 1325.82 count·s, respectively. These unbalanced, very small subsets cannot support inferential or validation claims.

At the 70% simulator setting, no protection trips occurred in any of the nine cells; descriptive mean IAE was 1527.38 count·s for fixed, 2343.55 for residual-plus-uncertainty, and 1349.83 for CRASI. Although this comparison is not confounded by protection trips, the post-inspection acquisition/analysis amendment prevents the 18-cell audit from serving as fresh validation. We therefore withdraw the direct audit from the evidentiary chain of the manuscript rather than reinterpret it as confirmatory evidence. The full audit trail and a fresh pre-outcome protocol for future direct validation are retained by the authors and are available upon reasonable request.

## 4. Discussion

### 4.1. Why Control Relevance Changes the Decision

Residual magnitude measures disagreement with the nominal predictor. CRASI modifies that evidence using common subtraction and heuristic operating-point relevance while retaining finite measurements and tracking bias. The observed benefit is conditional on those combined design choices and the surrogate domain. The actual command diagnostic, all-episode activity, matched residual comparison, and simultaneous-fault tests limit the interpretation: saturation can make the relevance proxy high while command impact is zero; a low gate can avoid a comparator-induced disturbance without correcting the underlying fault; and correlated anomalies can suppress both actions.

Current under-read illustrates the distinction. CRASI and fixed control have identical original IAE of 794.5 count·s, whereas residual + uncertainty has 944.9 and the no-relevance ablation 934.1. The revision diagnostics quantify whether CRASI acts at all and whether relevance based on model prediction changes the decision. This tracking comparison cannot be interpreted as current-fault correction or improved safety, particularly because the faulty current estimate can suppress relevance while true current exceeds the soft limit.

Encoder bias provides stronger evidence of active finite accommodation: the bias tracker and nonzero gate act frequently, and CRASI improves on fixed control in the original held-out domain. Even here, the matched residual observer must be considered before attributing the entire crossover to relevance. The nominal-domain residual result remains better than CRASI, demonstrating the trade-off between aggressive compensation under model match and retained feedback under mismatch.

### 4.2. Relationship to Existing FTC and Control-Aware Diagnosis

Observer-based current- and speed-sensor FTC methods commonly reconstruct faulty measurements after residual or statistical diagnosis [6,7,8,9]. Recent examples strengthen parameter robustness with a dedicated EKF [10], combine observer banks with experimentally validated control reconfiguration [11], and demonstrate real-time rotor-flux-observer diagnosis and sensorless fallback [13]; sensorless PMSM FTC has likewise integrated electrical- and sensor-fault accommodation in one architecture [12]. These methods are indispensable when continued operation requires a replacement signal, particularly for hard outages. CRASI addresses a complementary decision problem: a finite residual can be real yet not worth acting on, or can be partly caused by plant shift. Robust M-estimation provides another principled response to contaminated innovations; we retain Huberś original paper as the foundational source [25] and cite a recent Huber-based multi-sensor filtering formulation for contemporary context [26]. Adaptive residual thresholds likewise aim to prevent model uncertainty from being mistaken for faults under modeling uncertainty [27]. The post-primary Huber comparison shows that robust innovation weighting removes much of the nuisance behavior of hard residual rejection. Performance differences remain across fault types, but this comparison does not isolate control relevance from CRASI’s finite-trust, bias-tracking, and other observer design elements.

The work is also related to control-aware fault detection. Qi et al. include control actions in learned dynamic surrogate models [15], and Du et al. jointly consider detection and closed-loop performance [14]. CRASI takes a lightweight embedded-oriented route: it modifies the *decision value* of residual evidence by an explicit control-relevance factor rather than training a high-capacity dynamic model. This sacrifices representational power but yields an interpretable mechanism suitable for low-resource controllers and transparent ablation.

Uncertainty calibration is likewise used conservatively. Recent uncertainty-aware diagnosis emphasizes calibrated uncertainty for trustworthy decisions [16], while conformal methods provide distribution-free uncertainty statements under stated assumptions and are increasingly being evaluated in industrial fault diagnosis and distribution-shifted time series [17,18,19]. Here, calibration defines healthy residual/error scales and initializes online uncertainty, but the study does not claim fault-conditional or domain-shift conformal validity. Indeed, the observed coverage loss under injected faults shows why such a claim would be inappropriate. This distinction is consistent with recent cross-asset residual-conformal monitoring, which treats nominal error budgets as operational calibration targets when distribution shift undermines exchangeability [20], and with physics-guided bearing diagnosis showing that false-alarm calibration does not guarantee diagnostic transfer under unit or speed variability [21]. CRASI addresses a different closed-loop question—whether sensor-specific evidence should influence the current control action—but the same principle applies: calibration, diagnosis, control consequence, and transferability should be reported separately.

### 4.3. What the Results Do Not Show

Several boundaries remain essential. The original two host 70% load constructions still extrapolate lower-load characterization and use a memoryless current map. The broad fault campaign remains host-side evidence. The revision-era direct Proteus audit is explicitly excluded from validation because its original strict acquisition protocol was not completed and its complete analysis required post-inspection gate amendments. Position and speed share one encoder and current affects an envelope rather than torque/flux feedback. General multi-fault robustness, current-loop/FOC transfer, and timing, supply, thermal, communication and actuator fault coverage remain unestablished.

Relevance is heuristic and saturation-sensitive; self-masking and simultaneous-fault blindness persist in the original design. Healthy-scale uncertainty loses coverage under faults and position uncertainty has no active command effect. All added revision studies are exploratory or descriptive. Historical partial-kernel sizes remain distinct from the new complete linked AVR firmware and observed scheduling measurements. The latter establish execution of the tested episodes, not measured stack bounds, WCET or deployment feasibility.

The evidence supports selective tracking comparisons in specified host surrogates. It does not establish fresh direct-simulation validation, externally verified prospective testing, universal relevance-driven accommodation, sensor-independent current protection, physical safety improvement, or deployment-level FTC.

### 4.4. Recommended Physical Validation Path

Fresh direct Proteus/HIL/physical validation and broader independent fault coverage remain necessary. To avoid repeating the post-inspection ambiguity identified in the revision-era audit, the next campaign must freeze controller parameters, acquisition gates, analysis criteria, protection-trip handling, endpoints and exclusions before any outcome inspection and should use an independently auditable repository timestamp or archival deposit. The authors have retained such a protocol template; it contains no acquired outcomes and is available upon reasonable request. Any redesigned protective envelope or different controller requires its own specification and evaluation; all trips, missing detections and adverse outcomes must be preserved.

## 5. Conclusions

CRASI combines heuristic relevance with common-evidence subtraction, finite trust retention, and bias tracking. In the original 2520-episode host-side campaign it improved held-out IAE versus residual + uncertainty for four finite fault conditions. Encoder bias also improved on fixed control, whereas drift and current bias were slightly adverse, under-read equaled fixed, and dropouts were effectively null. All-episode diagnostics and matched observers restrict attribution: avoidance of aggressive-comparator degradation is distinct from active fault accommodation; drift does not support attributing the full gain to relevance. Position-uncertainty derating was inactive, and no hard-safety superiority is demonstrated.

Existing Huber and alternate-load studies and the revision diagnostics are exploratory. The revision-era direct Proteus audit is not used as validation because the complete allocation required post-inspection analysis-gate amendments and several 50% cells were affected by protection trips. Comparator dependence, simultaneous-fault blindness and current self-masking remain material. Fresh pre-specified direct Proteus/HIL/physical validation, validation in current-controlled/FOC architectures, and broader full-firmware timing/resource tests are required before safety or deployment claims.

## Figures and Tables

**Table 1:** Primary held-out results. IAE values are means over 30 paired episodes. CI is the 95% paired-bootstrap interval for proposed minus residual + uncertainty IAE. $p_{H}$ is Holm-adjusted across the six fault scenarios for the IAE endpoint.

Scenario	Fixed IAE	Residual + U	Proposed	Improvement	Paired Difference [95% CI]
Encoder bias	1192.5	1694.2	1057.3	37.6%	−636.9 [−786.8,−484.9]
Encoder drift	1018.6	1289.8	1033.0	19.9%	−256.8 [−352.5,−159.9]
Encoder dropout	1146.0	1146.0	1146.0	−0.001%	0.014 [−0.119,0.144]
Current magnitude bias	901.1	945.0	903.3	4.4%	−41.8 [−46.4,−36.7]
Current gain under-read	794.5	944.9	794.5	15.9%	−150.4 [−163.7,−136.2]
Current dropout	918.0	918.0	918.0	−0.001%	0.005 [−0.046,0.063]

Holm-adjusted $p_{H}$: encoder bias $1.0\times 10^{-5}$; encoder drift $7.84\times 10^{-4}$; current magnitude bias $1.0\times 10^{-5}$; current gain under-read $1.0\times 10^{-5}$; both dropouts 0.881.

**Table 2:** Held-out paired IAE differences: CRASI minus comparator [95% episode-bootstrap CI], count·s, n=30. Negative favors CRASI. Fixed and Huber use the archived paired runs; matched residual is added in revision. All intervals here are secondary/exploratory.

Scenario	Fixed	Matched Residual	Adaptive Huber
Healthy	0.00 [0.00,0.00]	−0.19 [−0.35,−0.05]	4.25 [3.65,4.85]
Encoder bias	−135.18 [−149.64,−120.74]	−11.15 [−23.37,−0.85]	−129.04 [−144.98,−112.87]
Encoder drift	14.41 [11.64,17.11]	11.11 [7.06,15.26]	20.41 [16.47,24.42]
Encoder dropout	0.00 [0.00,0.00]	−0.19 [−0.39,0.03]	−8.98 [−9.58,−8.40]
Current bias	2.12 [1.53,2.72]	−6.38 [−7.81,−4.99]	−46.30 [−54.13,−38.28]
Current under-read	0.00 [0.00,0.00]	−139.64 [−152.26,−126.63]	−137.62 [−150.01,−124.80]
Current dropout	0.00 [0.00,0.00]	−0.11 [−0.27,0.03]	3.10 [2.52,3.64]

**Table 3:** Correct fault-window gate/trust activity (means of 30 episode summaries). Action is $g_{j}>0$ and rejection is $w_{j}<0.25$; CRASI finite rejection is zero by construction. Encoder or current channel is selected according to the fault.

Fault	CRASI Action (%)	CRASI Trust	Residual + U Action (%)	Residual + U Trust	Residual + U Rejection (%)
Encoder bias	97.97	0.5639	47.80	0.5224	47.74
Encoder drift	0.00	1.0000	0.00	1.0000	0.00
Current bias	8.80	0.9627	100.00	0.2740	39.52
Current under-read	0.00	1.0000	9.79	0.9041	9.51

**Table 4:** Range of mean IAE percentage changes versus residual + uncertainty across tested one-factor settings (negative favors CRASI). The least favorable setting and its 95% bootstrap interval are shown. These ranges are not global parameter bounds.

Fault	Best (%)	Least Favorable (%)	Least Favorable Setting	95% CI (%)
Encoder bias	−35.95	−23.40	Floor = 0	[−34.11, −11.01]
Encoder drift	−19.81	−10.34	Floor = 0	[−14.70, −5.68]
Current under-read	−15.77	−15.77	β = 0.25	[−16.55, −14.90]

**Table 5:** Synthetic guard stress under current under-read; reference scale 1.4, 30 episodes per row. Peak is the maximum true absolute current across complete episodes. Fractions are means of episode fractions. Conservative relevance equals model relevance in these tracking/guard outcomes.

Current Multiplier	Method	Worst Current (A)	Soft (%)	Hard (%)	Position Guard (%)
2	CRASI	0.6794	8.58	0.00	5.10
2	Model relevance	0.6794	8.58	0.00	5.09
2	Fixed	0.6794	8.58	0.00	5.10
2	Residual + U	0.6794	8.90	0.00	4.40
3	CRASI	1.0041	9.42	7.44	5.29
3	Model relevance	1.0041	9.43	6.88	5.16
3	Fixed	1.0041	9.42	7.44	5.29
3	Residual + U	1.0041	9.11	8.61	4.30

## Data Availability

The data and supporting analysis records underlying the findings of this study are available from the corresponding authors upon reasonable request.

## References

[1] Isermann R. (2005). Model-based fault-detection and diagnosis—Status and applications. Annu. Rev. Control.

[2] Ding S.X. (2013). Model-Based Fault Diagnosis Techniques: Design Schemes, Algorithms and Tools.

[3] Blanke M., Kinnaert M., Lunze J., Staroswiecki M. (2016). Diagnosis and Fault-Tolerant Control.

[4] Chandra M.V.S.S., Mohapatro S. (2024). Sensor fault tolerant control in modern and green power applications: A review. IEEE Trans. Instrum. Meas..

[5] Dutta K.K., Devanshu A. (2026). Evolution of fault-tolerant control in induction motors: From model-based to data-driven intelligence. IETE Tech. Rev..

[6] Azzoug Y., Pusca R., Sahraoui M., Ameid T., Romary R. (2023). Experimental validation of current sensors fault detection and tolerant control strategy for three-phase permanent magnet synchronous motor drives. Machines.

[7] Zhang F., Gao S., Zhang W., Li G., Zhang C. (2024). Current sensor fault-tolerant control strategy for speed-sensorless control of induction motors based on sequential probability ratio test. Electronics.

[8] Tran C.D., Kuchar M., Sotola V., Nguyen P.D. (2024). Fault-tolerant control based on current space vectors against total sensor failures. Sensors.

[9] Adamczyk M., Orłowska-Kowalska T. (2024). Current sensor fault-tolerant control based on modified Luenberger observers for safety-critical vector-controlled induction motor drives. Bull. Pol. Acad. Sci. Tech. Sci..

[10] Miniach M., Orłowska-Kowalska T. (2025). Innovative extended Kalman filter in a coherent system for detection and compensation of various current sensor faults in the induction motor drive. IEEE Trans. Ind. Electron..

[11] Venghi L.E., Aguilera F., de la Barrera P.M., De Angelo C.H. (2026). Sensors and open-switch FDI of induction motor drives based on control reconfiguration. Control Eng. Pract..

[12] Zhang Y., Wang X., Kong L., Lu L., Wang Z., Mao Y. (2025). Fault-tolerant control of common electrical faults and sensor faults in sensorless PMSM drives. IEEE Trans. Power Electron..

[13] Tran C.D., Kuchar M., Sotola V., Nguyen P.D. (2026). Sensor fault diagnosis strategy based on rotor flux observers in three-phase induction motor drive. Sci. Rep..

[14] Du Y., Budman H., Duever T.A. (2016). Integration of fault diagnosis and control based on a trade-off between fault detectability and closed loop performance. J. Process Control.

[15] Qi M., Jang K., Cui C., Moon I. (2023). Novel control-aware fault detection approach for non-stationary processes via deep learning-based dynamic surrogate modeling. Process Saf. Environ. Prot..

[16] Lin Y.-H., Li G.-H. (2024). Uncertainty-aware fault diagnosis under calibration. IEEE Trans. Syst. Man Cybern. Syst..

[17] Angelopoulos A.N., Bates S. (2023). Conformal prediction: A gentle introduction. Found. Trends Mach. Learn..

[18] Heddoub A., Diallo A.R., Homri L., Dantan J.-Y., Siadat A. (2025). Uncertainty-aware fault diagnosis with conformal prediction. IFAC-PapersOnLine.

[19] Zhang R., Zhou P. (2025). Uncertainty quantification based on conformal prediction for industrial time series with distribution shift. IEEE Trans. Ind. Inform..

[20] Haneef Z., Umar M., Saleem F., Ullah I., Aqeel K., Siddique M.F. (2026). Uncertainty-calibrated residual conformal monitoring of wind turbine SCADA data for cross-asset anomaly detection under distribution shift. Information.

[21] Siddique M.F., Wisal M., Umar M., Ali S., Janjua M.M., Khalid H., Haneef Z., Akhtar N. (2026). A physics-guided statistical framework for bearing fault diagnosis under unit variability and rotational speed uncertainty. Machines.

[22] Microchip Technology Inc. ATmega640/1280/1281/2560/2561 Complete Datasheet and ATmega2560 Product Page. https://www.microchip.com/en-us/product/atmega2560.

[23] Allegro MicroSystems ACS712 Fully Integrated Hall-Effect-Based Linear Current Sensor IC Datasheet. https://www.allegromicro.com/en/products/sense/current-sensor-ics/integrated-current-sensors/acs712.

[24] STMicroelectronics (2023). L298 Dual Full-Bridge Driver, DS0218 Rev. 5. https://www.st.com/en/motor-drivers/l298.html.

[25] Huber P.J. (1964). Robust estimation of a location parameter. Ann. Math. Stat..

[26] Park J.-S., Kim G.-H., Kwon H.-H., Kim J.-H. (2025). Huber-based asynchronous fusion filter for robust multi-rate sensor processing. Int. J. Control Autom. Syst..

[27] Intriago G., Zhang Y. (2024). Adaptive fault detection for grid-forming inverters. IEEE Access.

[28] Holm S. (1979). A simple sequentially rejective multiple test procedure. Scand. J. Stat..

